# Intrinsic MyD88-Akt1-mTOR Signaling Coordinates Disparate Tc17 and Tc1 Responses during Vaccine Immunity against Fungal Pneumonia

**DOI:** 10.1371/journal.ppat.1005161

**Published:** 2015-09-14

**Authors:** Som Gowda Nanjappa, Nydiaris Hernández-Santos, Kevin Galles, Marcel Wüthrich, M. Suresh, Bruce S. Klein

**Affiliations:** 1 Department of Pediatrics, University of Wisconsin School of Medicine and Public Health, Madison, Wisconsin, United States of America; 2 Department of Pathobiological Sciences, School of Veterinary Medicine, University of Wisconsin, Madison, Wisconsin, United States of America; 3 Department of Internal Medicine, University of Wisconsin School of Medicine and Public Health, Madison, Wisconsin, United States of America; 4 Department of Medical Microbiology and Immunology, University of Wisconsin School of Medicine and Public Health, Madison, Wisconsin, United States of America; The University of Texas at San Antonio, UNITED STATES

## Abstract

Fungal infections have skyrocketed in immune-compromised patients lacking CD4^+^ T cells, underscoring the need for vaccine prevention. An understanding of the elements that promote vaccine immunity in this setting is essential. We previously demonstrated that vaccine-induced IL-17A^+^ CD8^+^ T cells (Tc17) are required for resistance against lethal fungal pneumonia in CD4^+^ T cell-deficient hosts, whereas the individual type I cytokines IFN-γ, TNF-α and GM-CSF, are dispensable. Here, we report that T cell-intrinsic MyD88 signals are crucial for these Tc17 cell responses and vaccine immunity against lethal fungal pneumonia in mice. In contrast, IFN-γ^+^ CD8^+^ cell (Tc1) responses are largely normal in the absence of intrinsic MyD88 signaling in CD8^+^ T cells. The poor accumulation of MyD88-deficient Tc17 cells was not linked to an early onset of contraction, nor to accelerated cell death or diminished expression of anti-apoptotic molecules Bcl-2 or Bcl-xL. Instead, intrinsic MyD88 was required to sustain the proliferation of Tc17 cells through the activation of mTOR via Akt1. Moreover, intrinsic IL-1R and TLR2, but not IL-18R, were required for MyD88 dependent Tc17 responses. Our data identify unappreciated targets for augmenting adaptive immunity against fungi. Our findings have implications for designing fungal vaccines and immune-based therapies in immune-compromised patients.

## Author Summary

Patients with AIDS, cancer or immune suppressive treatments are vulnerable to infection with invasive fungi. We have found that even when helper CD4 T cells are profoundly reduced in a mouse model that mimics this defect in AIDS, other remaining T cells are capable of mounting vaccine immunity against a deadly fungal infection, and they do so by producing the powerful, soluble product, IL-17. It has been widely believed that the activation and instruction of such cells, called Tc17 cells, is governed by another population of immune cells in the body, but we have found here that pathways within these Tc17 cells themselves mediate their activation and ability to produce the IL-17 needed for resistance to infection. We have also identified elements of the circuitry controlling this pathway—elements called MyD88, Akt1 and mTOR—and found that they control the production of IL-17 and not other products such as IFN-γ often produced by these cells. Further, we determined that this circuitry controls the development of Tc17 cells by regulating their ability to divide and expand. Thus, in a mouse model of vaccination against lethal fungal pneumonia caused by *Blastomyces dermatitidis*, we uncovered an important cellular arsenal that can be recruited to bolster resistance against a fungal infection, and identified novel ways in which the cells develop and expand into potent killers of fungi.

## Introduction

The rising incidence rate of life threatening fungal infections in immune-deficient hosts requires preventive measure in *at risk* individuals. CD4^+^ T cells are the primary effector cells that control fungal infections in healthy hosts, and their loss in lymphopenic patients necessitates targeting residual immune subsets to elicit antifungal immunity. We previously showed in a mouse model of lethal fungal pneumonia that, even in the absence of CD4^+^ T cell help, vaccine-induced CD8^+^ T cells could differentiate and expand into cytokine producing cells, persist as long-lasting memory cells, and mediate sterilizing immunity [1]. Antifungal CD8^+^ T cells that produce IL-17A are indispensable in this model. In contrast, CD8^+^ T cells that produce type I cytokines (IFNγ, TNFα or GM-CSF) contribute to vaccine immunity, but are expendable [2,3]. A deeper understanding of the elements required to elicit CD8^+^ T cell responses will be required to catalyze the development of rationally designed anti-fungal vaccines.

T cell respond to antigen in three distinct phases: in the expansion phase, upon recognition of cognate antigen, T cells undergo rapid proliferation and differentiation into effectors; in the contraction phase, ~90% of effectors T cells die by apoptosis; and in the memory phase, the remaining 10% of effector T cells differentiate into long-lasting memory cells. Hence, in general, the magnitude of expansion and survival of effector cells will dictate protective immunity [4]. The inflammatory milieu influences the quality and quantity of effector T cells. For example, a lack of type I interferon signaling abrogates clonal expansion of CD8^+^ T cells due to reduced survival, whereas enhanced inflammation exaggerates terminal differentiation and apoptosis [5,6]. Among other factors, cytokines regulate differentiation of T cells into distinct subsets that express prototypic transcription factors and signature cytokines. For Th17 cell responses, different combinations of cytokines including IL-6, TGFβ, IL-1, IL-21 and IL-23 have been implicated in differentiation *in vitro* and *in vivo* [7,8].

CD8^+^ T cell responses are typically associated with defense against intracellular pathogens and tumors by mechanisms that are largely dependent on IFNγ, granzyme, and perforin. CD8^+^ T cells control fungal infections chiefly by secretion of proinflammatory cytokines such as IFN-γ, TNF-α, and GM-CSF that activate phagocytes to kill fungi [9]. A distinct subset of IL-17A producing CD8^+^ T cells, Tc17 cells, also play a role in defense against infections and tumors. Elimination of Tc17 cells is associated with progressive SIV/HIV infection [10–12] and Tc17 cells are protective against vaccinia and influenza virus infections [13,14] and tumors [15,16]. Likewise, we have found that Tc17 cells are indispensable for vaccine-induced protection against fungal pneumonia [2]. Differentiation of Tc17 cells requires TGFβ and IL-6 or IL-21 [17]; IL-23 signaling has been shown to promote pathogenic Tc17 cells [18]. IRF4 facilitates Tc17 responses by transcriptionally activating RORγt and RORα and repressing EOMES and FOXP3, while IRF3 inhibits Tc17 programming by altering RORγt promoter binding [19,20]. The molecular switch that regulates initial programming of Tc1 and Tc17 responses under similar *in vivo* ‘inflammatory milieu’ is poorly understood.

MyD88, a signaling adaptor for TLRs and IL-1R family members in myeloid cells, is critical for innate and adaptive immunity [21]. MyD88 signaling activates macrophages and DCs, elicits production of proinflammatory cytokines and promotes antigen presentation to initiate adaptive immune responses during viral, bacterial and parasitic infections [22]. Impaired MyD88 signaling increases susceptibility to fungal infections such as candidiasis, cryptococcosis, aspergillosis, paracoccidioidosis, pneumocystis and coccidioidomycosis [23–25]. Conversely, bolstering MyD88 signaling in dendritic cells improves resistance to aspergillosis [26,27]. Thus, MyD88 signaling in myeloid cells plays an integral role in immunity against fungal infections. However, the T cell-intrinsic role of MyD88 in adaptive immune responses to fungal infections has not been defined.

In experimental *Toxoplasma gondii* infection, T cell expression of MyD88 is required for Th1 mediated resistance [28]. This Th1 response is independent of IL-1R and IL-18R, implying a role for TLRs in orchestrating MyD88-mediated T cell responses to *T*. *gondii*. Toll-like Receptor 2 signaling in CD4^+^ T cells is known to promote Th17 responses *in vitro* [29] and regulate the pathogenesis of autoimmunity in a model of experimental autoimmune encephalitis (EAE). During LCMV infection, IFNγ-producing CD8^+^ T cells (Tc1 cells) require intrinsic MyD88 signals for differentiation and survival [30,31]. The importance of intrinsic MyD88 signals for the development of Tc17 cells that confer resistance against microbes including fungi remains poorly understood.

We have reported that IL-17-producing CD8^+^ T cells are indispensible in mediating vaccine immunity against fungal pneumonia in CD4^+^ T cell deficient mice [2]. In the current study, we investigated the underlying mechanisms that enable the priming and development of these potent vaccine effectors. Here, using a mouse model of vaccination against lethal fungal pneumonia caused by *Blastomyces dermatitidis*, we show that T cell-intrinsic MyD88 signals are required for Tc17 cell responses and immunity. In contrast, Tc1 responses are relatively spared in the absence of such signals. Unlike the situation for anti-viral CD8^+^ T cells, poor accumulation of anti-fungal Tc17 cells is not linked to accelerated death or reduced expression of anti-apoptotic molecules Bcl-2/Bcl-xL. Instead, the poor accumulation is due to impaired proliferation that is mediated via Akt1 through the mTOR pathway. Moreover, we show that IL-1R and TLR2, and not IL-18R, are the upstream sensors and signaling receptors that initiate these anti-fungal Tc17 cell responses. Thus, we describe the novel contribution of intrinsic MyD88 signals in Tc17 cells during the development of anti-fungal immunity, and the role of the AkT1-mTOR axis in fostering sustained proliferation of these cells and establishment of Tc17 memory and immunity in CD4^+^ T cell deficient hosts.

## Results

### Intrinsic MyD88 expression is required for Tc17 responses

**Fig 1:**
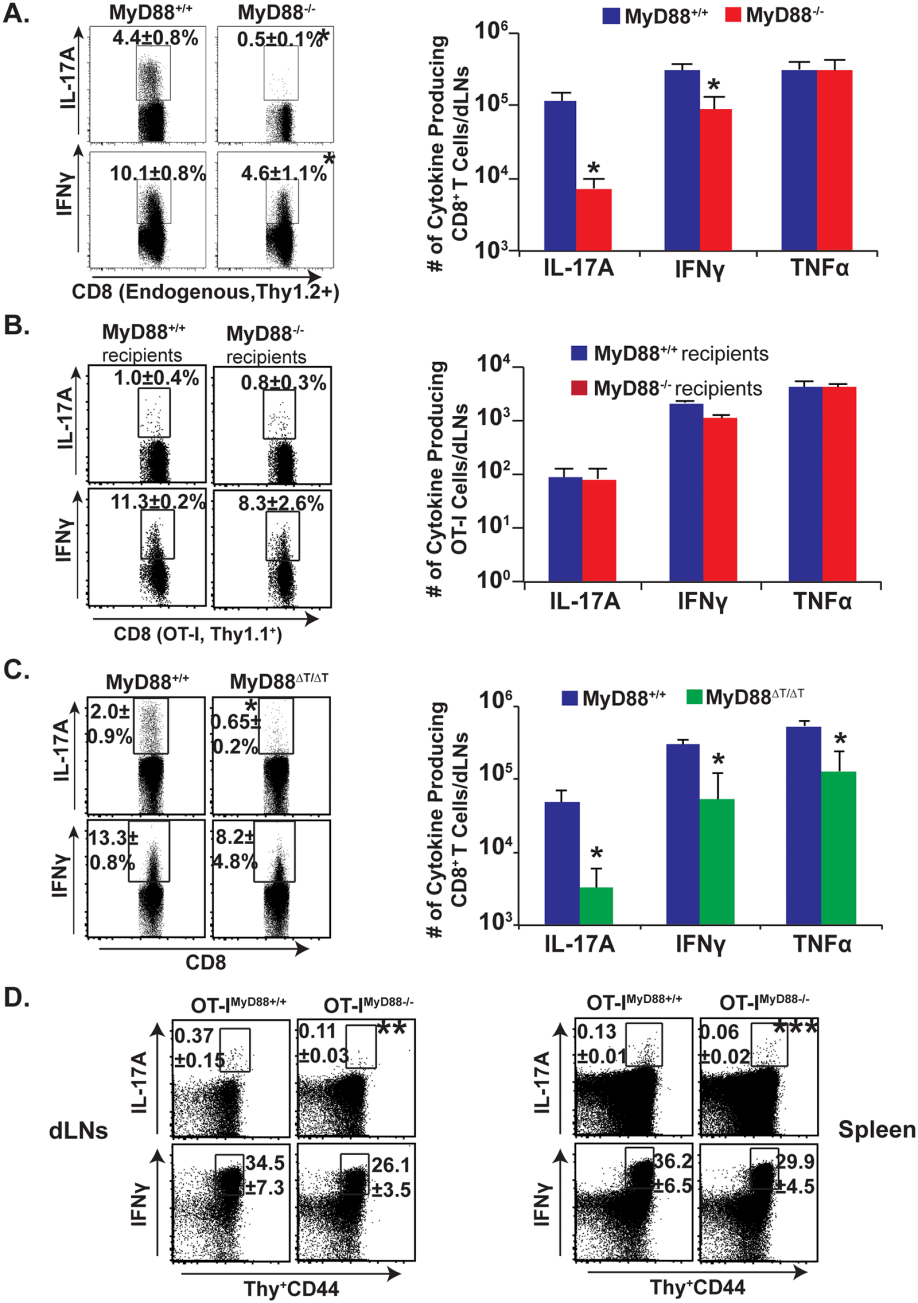
Intrinsic MyD88 signaling regulates Tc17 cell responses. Naïve purified OT-I cells (10^6^) were adoptively transferred into naïve congenic wild-type (WT) or MyD88^-/-^ mice **(A & B)**. Naïve Thy1.1^+ve^ OT-I^wt^ or OT-I^myd88-/-^ T cells were transferred into naïve congenic Thy1.2^+ve^ WT mice **(D)**. Mice were CD4^+^ T cell depleted and vaccinated with OVA-expressing (OT-I) #55 yeast **(A, B, D)** or #55 yeast (**C**). Draining LNs and spleens were harvested after 2–3 weeks to analyze percent and total number of cytokine producing CD8^+^ CD44^hi^ T cells by flow cytometry. CD4^+^ T cells were depleted throughout the experiment. Values are mean ± SD of 4–5 mice/group. *P≤0.05. Data is representative of 2–4 independent experiments.

We initially investigated the general requirement of MyD88 signaling for Tc17 responses following fungal vaccination. We adoptively transferred OT-I cells into naïve congenic wild-type and MyD88^-/-^ mice, and vaccinated the animals with attenuated recombinant *Blasomyces* yeast expressing the OVA epitope SIINFEKL. On day 18 post-vaccination, following *ex vivo* restimulation with anti-CD3/CD28 antibodies, we first analyzed the percentage and total number of endogenous Tc17 and Tc1 cells that lack MyD88 by gating on activated Thy1.2^+ve^ CD8^+^ T cells (CD44^hi^) (Fig 1A). The endogenous, IL-17 producing CD8^+^ T cells in MyD88^-/-^ mice were severely blunted in the draining lymph nodes (dLNs) and spleen, whereas IFN-γ producing cells were largely spared (8.8 fold vs. 2.2 fold reduction, respectively). MyD88 signals therefore are required to promote the generation of Tc17 cell responses after fungal vaccination. To dissect the intrinsic vs. extrinsic requirement for MyD88, we analyzed the transferred, wild-type, OT-I cells bearing a distinct, congenic Thy1.1 marker. Surprisingly, IL-17A^+^ and IFNγ^+^OT-I responses were largely intact in both the wild-type and MyD88^-/-^ recipients (Fig 1B). Thus, intrinsic MyD88 signaling is involved in CD8^+^ T cell responses, especially for the Tc17 subset.

To study the intrinsic role of MyD88 in Tc17 cells, we pursued further approaches. First, we purified CD8^+^ T cells from naïve wild-type and MyD88^-/-^ mice and transferred them into naïve TCRα^-/-^ mice (S1A Fig). Recipients were vaccinated and challenged by the pulmonary route to assess recall responses in the lung, which are reminiscent of vaccine responses in the dLNs. Vaccinated TCRα^-/-^ hosts that received wild-type CD8^+^ T-cells had pronounced Tc17 cell responses compared to unvaccinated recipients (S1B and S1C Fig). Vaccinated TCRα^-/-^ hosts that received MyD88^-/-^ CD8^+^ T-cells had significantly lower Tc17 responses vs. the recipients of wild-type cells (~10 fold, p≤0.05). These data support the hypothesis that intrinsic MyD88 signaling is required for Tc17 more than Tc1 responses to a fungal infection (Fig 1A and 1B and S1 Fig).

In an alternative approach, we confirmed an intrinsic role of MyD88 for CD8^+^ T cell responses by using MyD88ΔT mice in which only T cells lack MyD88. After vaccination and analysis of the dLNs, Tc17 cells were significantly impaired in MyD88ΔT mice vs. wild-type mice, whereas Tc1 cells were relatively spared (Fig 1C; p≤0.05). In yet another approach, to assess antigenic specificity and exclude possible developmental T-cell repertoire anomalies in MyD88ΔT mice, we tested OT-I^myd88-/-^ mice. We transferred OT-I cells into congenic recipients, vaccinated with recombinant OVA yeast and analyzed SIINFEKL-specific Tc17 and Tc1 responses. OT-I cells lacking MyD88 produced significantly less IL-17A compared to wild-type OT-I cells in the dLNs and spleen (Fig 1D; p≤0.05). MyD88 signaling was relatively dispensable for IFN-γ responses. *In vitro* studies with OT-I cells and OVA-vaccine yeast illustrated the non-redundant role of TCR signaling in fungal-induced Tc17 responses, and studies with naïve CD8^+^ T cells illustrated the cell intrinsic role of MyD88 for Tc17 cell responses (S2A and S2B Fig). Thus, intrinsic MyD88 signals preferentially affect Tc17 over Tc1 responses after fungal vaccination.

### Intrinsic MyD88 expression in CD8^+^ T cells is required for fungal resistance

**Fig 2:**
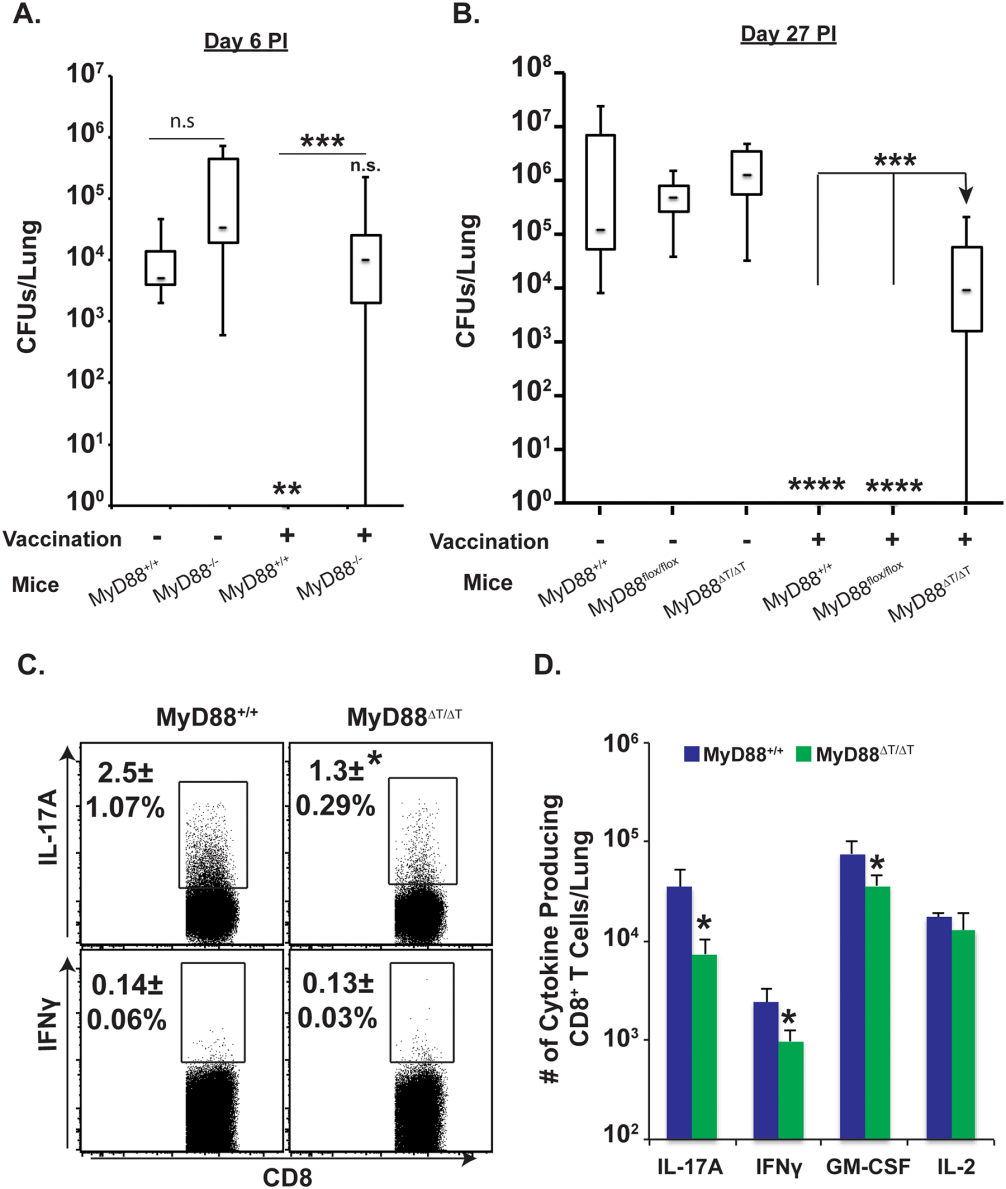
Intrinsic MyD88 signaling in CD8 T cells is required for vaccine immunity. **(A & B)** CD4^+^ T-cell depleted naïve mice were vaccinated with live **(A)** or heat-killed **(B)** #55 yeast (B). Vaccinated mice were challenged with virulent #26199 yeast, lungs were harvested for CFU analysis on indicated days, and data are shown in whisker plots **(A & B**; N = 7–14 mice/group). Lungs were harvested 4 days post-challenge to enumerate percentage **(C)** and total number of cytokine-producing CD8^+^ T cells **(D)** by flow cytometry. CD4^+^ T cells were depleted throughout experiment. Values are the mean ± SD of 5 mice/group. *p≤0.05, **p≤0.01, ***p≤0.001 and ****p≤0.0001. Data is representative of 2 independent experiments.

Previously, we showed that Tc17 cells were necessary for vaccine immunity in the absence of CD4^+^ T cells [2]. Here, we explored the functional role of MyD88 signaling in vaccine resistance of CD4^+^ T cell depleted mice. Unvaccinated wild-type mice failed to control pulmonary infection and harbored ~4 log cfu of yeast in their lungs, whereas vaccinated mice acquired sterilizing immunity (Fig 2A). Unvaccinated MyD88^-/-^ mice have a slightly higher fungal burden than unvaccinated wild-type mice indicating MyD88 promotes innate resistance in the lung. However, vaccinated MyD88^-/-^ mice failed to acquire immunity and exhibited a fungal burden similar to unvaccinated wild-type mice (Fig 2A). Thus, MyD88 signaling is essential for vaccine immunity.

To assess a cell-intrinsic role of MyD88 for vaccine-induced CD8^+^ T cell immunity, we vaccinated MyD88ΔT mice. Vaccinated MyD88ΔT mice had a significantly higher fungal burden than vaccinated control mice (Fig 2B). Vaccinated MyD88ΔT mice did have a lower fungal burden (~1 log) than unvaccinated controls, suggesting contributions to vaccine resistance by IFN-γ, TNFα, GM-CSF and IL-17A that are MyD88 independent. To correlate the resistance phenotype with cellular infiltration of cytokine producing CD8^+^ T cells, we harvested lungs 4 days after challenge (peak of cell influx) and analyzed cells by flow cytometry. The percentage of IL-17A^+^ CD8^+^ T cells in the lungs was significantly lower in vaccinated MyD88ΔT mice than controls (Fig 2C; p≤0.05). The total numbers of IL-17A^+^, IFNγ^+^ and GM-CSF^+^ CD8^+^ T cells also were significantly lower in vaccinated MyD88ΔT mice than vaccinated controls, with a greater impact on Tc17 cells than Tc1 cells (Fig 2D; ~8-fold vs. 2-fold, respectively). Thus, impaired immunity in vaccinated MyD88ΔT mice was correlated with poor influx and/or accumulation of cytokine-producing CD8^+^ T cells in lungs, reflecting impaired vaccine responses in dLNs and spleens (Figs 1C and 2). Collectively, these data suggest that intrinsic expression of MyD88 is required for vaccine-induced CD8^+^ T cell immunity and protective Tc17 cell responses.

### Intrinsic expression of MyD88 is required for the sustained expansion of Tc17 cells following vaccination

**Fig 3:**
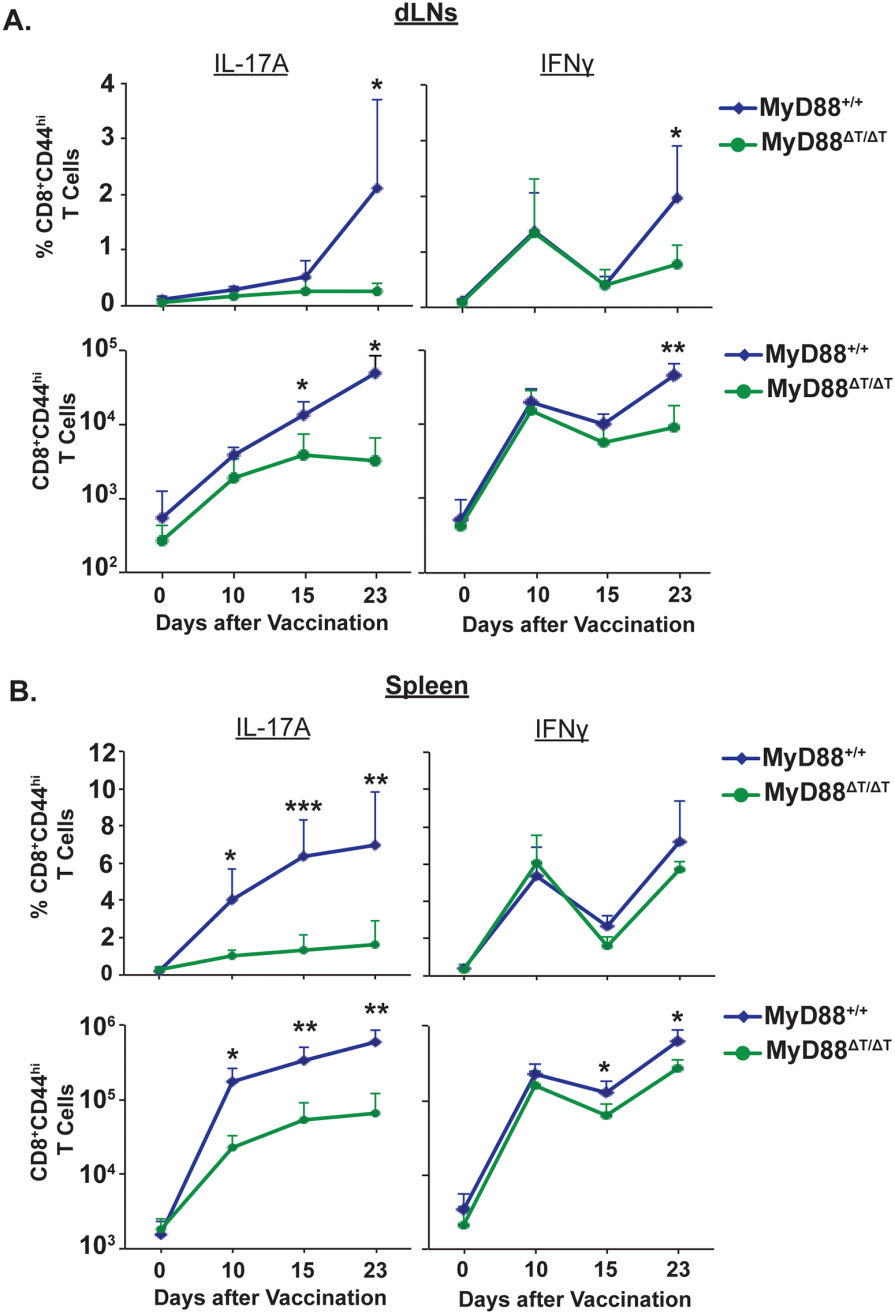
Kinetics of Tc17 and Tc1 cell responses in vaccinated mice. Naïve WT and MyD88ΔT mice were vaccinated and on indicated days. dLNs **(A)** and spleens **(B)** were harvested to analyze percent and total number of IFNγ^+^ and IL-17A^+^ CD8^+^ T cells. CD4^+^ T cells were depleted throughout the experiment. Data are mean ± SD of N = 4–5 mice/group. *P≤0.05.

In Fig 1, we investigated the intrinsic role of MyD88 signaling by analyzing CD8^+^ T cell responses approximately 3 weeks after vaccination. Here, we asked whether intrinsic MyD88 signaling affects CD8^+^ T cell responses during the early or late stages of expansion. These phases of expansion include priming, differentiation and proliferation of effector CD8^+^ T cells. To analyze the kinetics of CD8^+^ T cell responses during these phases, we vaccinated mice and assessed responses in the dLNs and spleens on days 0 (naïve), 10, 15 and 23. As early as day 10, the percentage and total numbers of IL-17A^+^ CD8^+^ T cells were significantly blunted in the spleens of MyD88ΔT vs. wild-type mice (Fig 3A and 3B). These impairments were evident in the dLNs only later, by 15 to 23 days post-vaccination, suggesting that differentiated Tc17 cells become effector cells and emigrate from dLNs. Tc1 cell responses also were reduced in the MyD88ΔT mice, but these impairments appeared later and were less pronounced than blunted Tc17 responses. Of note, in the early stages of expansion, the activation (CD44^hi^) of total CD8^+^ T cells was less impaired in MyD88ΔT mice, suggesting that intrinsic MyD88 signaling preferentially affects Tc17 cell responses (S3A Fig). Tc17 cells produced significantly less IL-17 on a per cell basis in MyD88ΔT vs. wild-type mice (mean fluorescence intensity: 9986±403 vs 6153±398; S3B Fig), suggesting that intrinsic MyD88 signaling shapes not only the quantity, but also the quality of Tc17 cells. We stained for RORγt, but found an insignificant difference between the two groups. Thus, MyD88 signaling in CD8^+^ T cells is required for optimal Tc17 cell expansion following fungal vaccination.

### Defective vaccine-induced Tc17 responses in MyD88ΔT mice is not due to augmented apoptosis or reduced CD43 and CD27 expression

**Fig 4:**
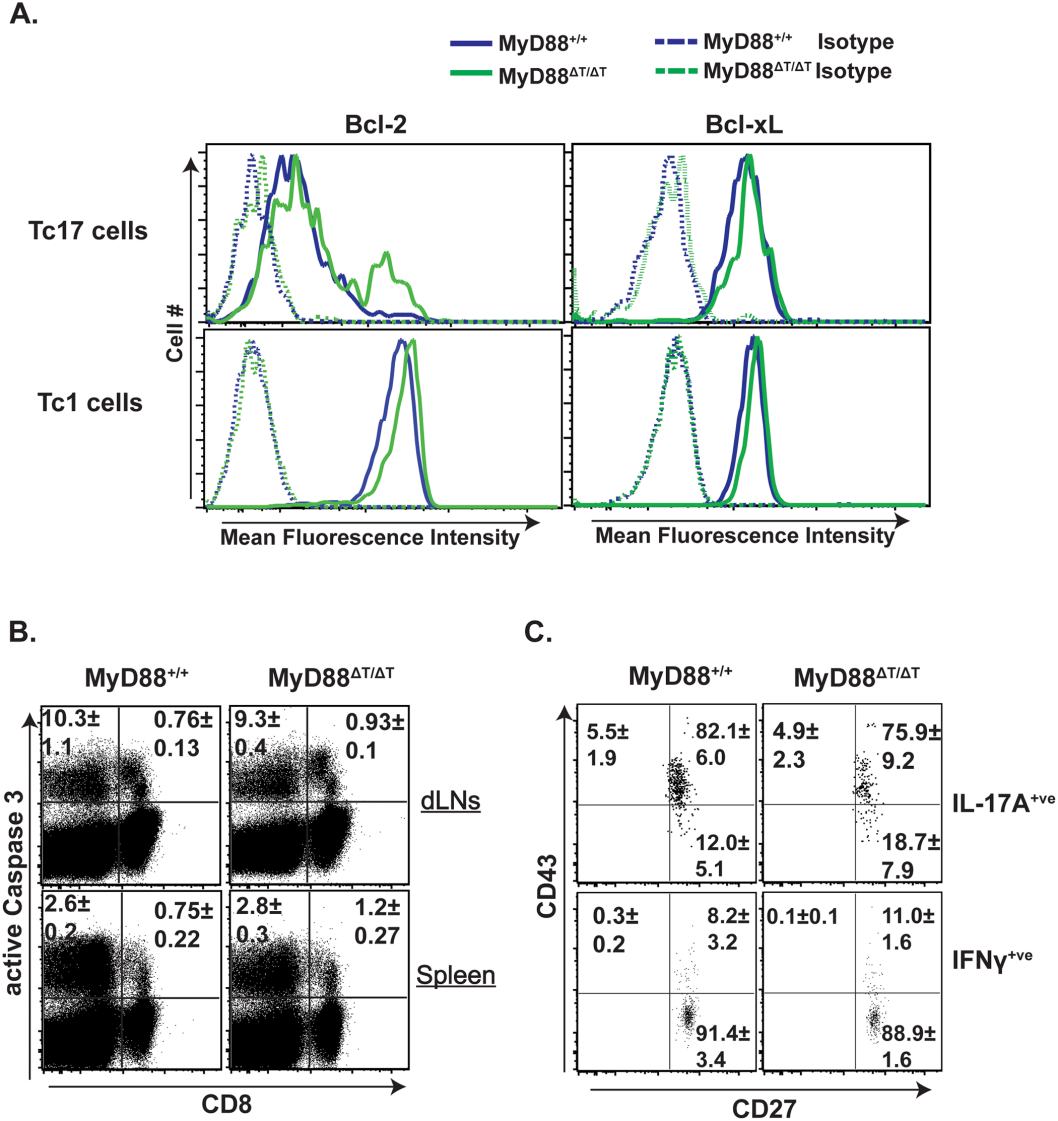
The role of intrinsic MyD88 for effector CD8^+^ T cell survival. Naïve WT and MyD88ΔT mice were CD4^+^ T cell depleted and vaccinated with strain #55. Two weeks later dLNs and spleen cells were collected, restimulated and stained for surface markers followed by intracellular staining using BD Perm/Fix and analyzed by flow cytometry. **A**. MFI of Bcl-2 and Bcl-xL expression in IFNγ^+^ (Tc1) and IL-17A^+^ (Tc17) cells. **B**. Active Caspase 3 expression. Numbers indicate percent active Caspase 3 expressing cells. **C**. Surface CD43 and CD27 expression in IL-17A^+^ and IFNγ^+^ cells. Values are the mean ± SD percentage; N = 4–5 mice/group. Data is representative of 2 independent experiments.

The net number of T cells during the expansion phase is governed by proliferation and apoptosis of effector cells. Bcl-2 and Bcl-xL play an important role in survival of effector CD8^+^ T cells [32]. We asked whether reduced expansion of Tc17 cells in MyD88ΔT mice is linked to the reduced expression of anti-apoptotic molecules Bcl-2 and Bcl-xL. The expression levels of Bcl-2 and Bcl-xL in Tc17 (and Tc1) cells were comparable in vaccinated wild-type and MyD88ΔT mice (Fig 4A). We also assessed active caspase 3 following *ex vivo* restimulation, and found no significant differences between the groups in either cytokine-producing cells or in total CD8^+^ T cells (Fig 4B). Finally, we stained effector CD8^+^ T cells with Annexin V to detect signs of early apoptosis and again found no difference. Thus, reduced Tc17 cell responses in the absence of MyD88 signaling are not due to either reduced survival or augmented death of effector CD8^+^ T cells.

Our previous work showed that CD43 expression is higher in Tc17 than in Tc1 cells [2]. CD43 signaling has a dichotomous role in effector CD8^+^ T cells; CD43 promotes expansion during the early phase of the T cell response, but augments apoptosis in the later phase [33]. Similarly, CD27 signaling is necessary for survival and/or proliferation of effector CD8^+^ T cells [34]. Therefore, we explored whether reduced accumulation of Tc17 cells induced by deficient MyD88 signaling was associated with decreased CD43 and CD27 expression. Fig 4C shows the frequency of CD43^+^ and CD27^+^ expression on Tc17 and Tc1 cells. As before, CD43 expression was higher on Tc17 than Tc1 cells, but there was no significant difference between cells from wild-type and MyD88ΔT mice. Likewise, expression levels of CD27 on Tc17 and Tc1 cells were comparable between the groups (Fig 4C). Thus, poor Tc17 cell accumulation in the absence of MyD88 is neither due to augmented apoptosis nor to blunted CD43 or CD27 receptor expression.

### MyD88 signaling in Tc17 cells is required for sustained proliferation during expansion

**Fig 5:**
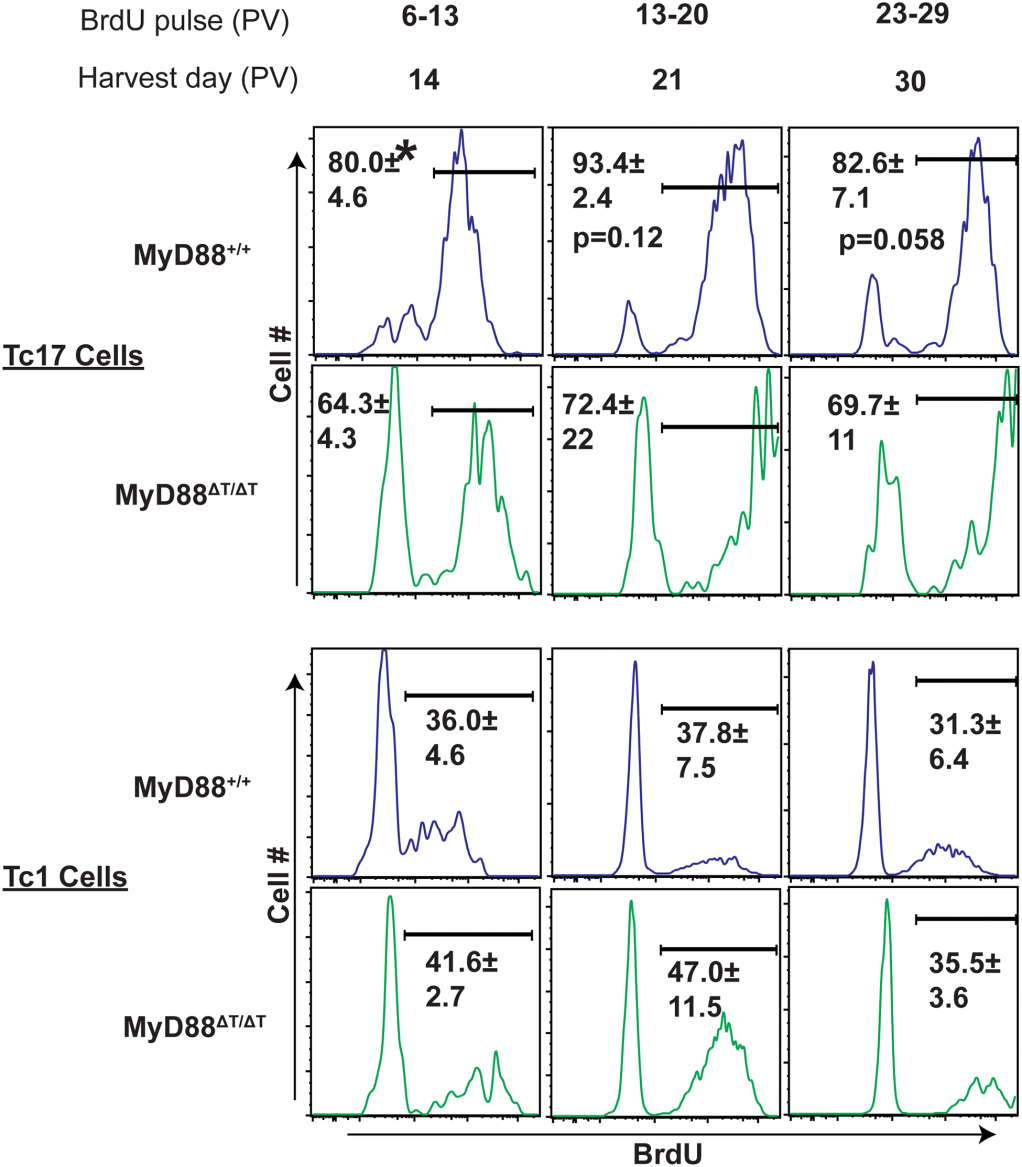
MyD88 signaling potentiates proliferation of Tc17 cells. Naïve WT or MyD88ΔT mice were CD4^+^ T cell depleted and vaccinated with strain #55. BrdU was pulsed through the drinking water on indicated interval days. Mice were sacrificed and dLNs were harvested at the end of each indicated pulse period. Cells were restimulated, stained for surface and intracellular cytokines before BrdU staining. Percent BrdU^+ve^ cells were analyzed by flow cytometry by gating on CD8^+^ IL-17- or IFNγ-producing cells. CD4^+^ T cells were depleted throughout the experiment. Values are percent mean ± SD of 4–5 mice/group. *P≤0.05. Data is representative of 3 independent experiments.

We evaluated whether MyD88 signaling regulated the proliferation of effector CD8^+^ T cells during the expansion and contraction phases of the T cell response. To evaluate CD8^+^ T cell proliferation, mice received BrdU for three intervals after vaccination (Fig 5). BrdU^+^ Tc17 and Tc1 cells were analyzed at the end of each period. Wild-type Tc17 cells in dLNs exhibited rapid proliferation by day 14 (80%), which peaked by day 21 (~93%) and showed contraction or memory transition by day 30 (~82%; Fig 5). Similar results were found in spleens, especially at day 30, where proliferation of Tc17 cells was dramatically reduced (S4 Fig). The proliferation of Tc17 cells in MyD88ΔT mice followed similar kinetics, however the percentage of BrdU^+^ cells was significantly lower on day 14 and remained lower at subsequent time points. Unlike the proliferation defect in Tc17 cell, the absence of MyD88 signaling did not significantly affect Tc1 proliferation (Fig 5). We also did not detect proliferation defects in MyD88-deficient, activated CD8^+^ (CD44^hi^) T cells during the early phases of expansion. Thus, MyD88 signaling sustained the proliferation of Tc17 cells, but not Tc1 cells, throughout the expansion phase, without exhibiting delayed expansion following fungal vaccination.

### MyD88 signaling mediates proliferation of Tc17 cells via mTOR

**Fig 6:**
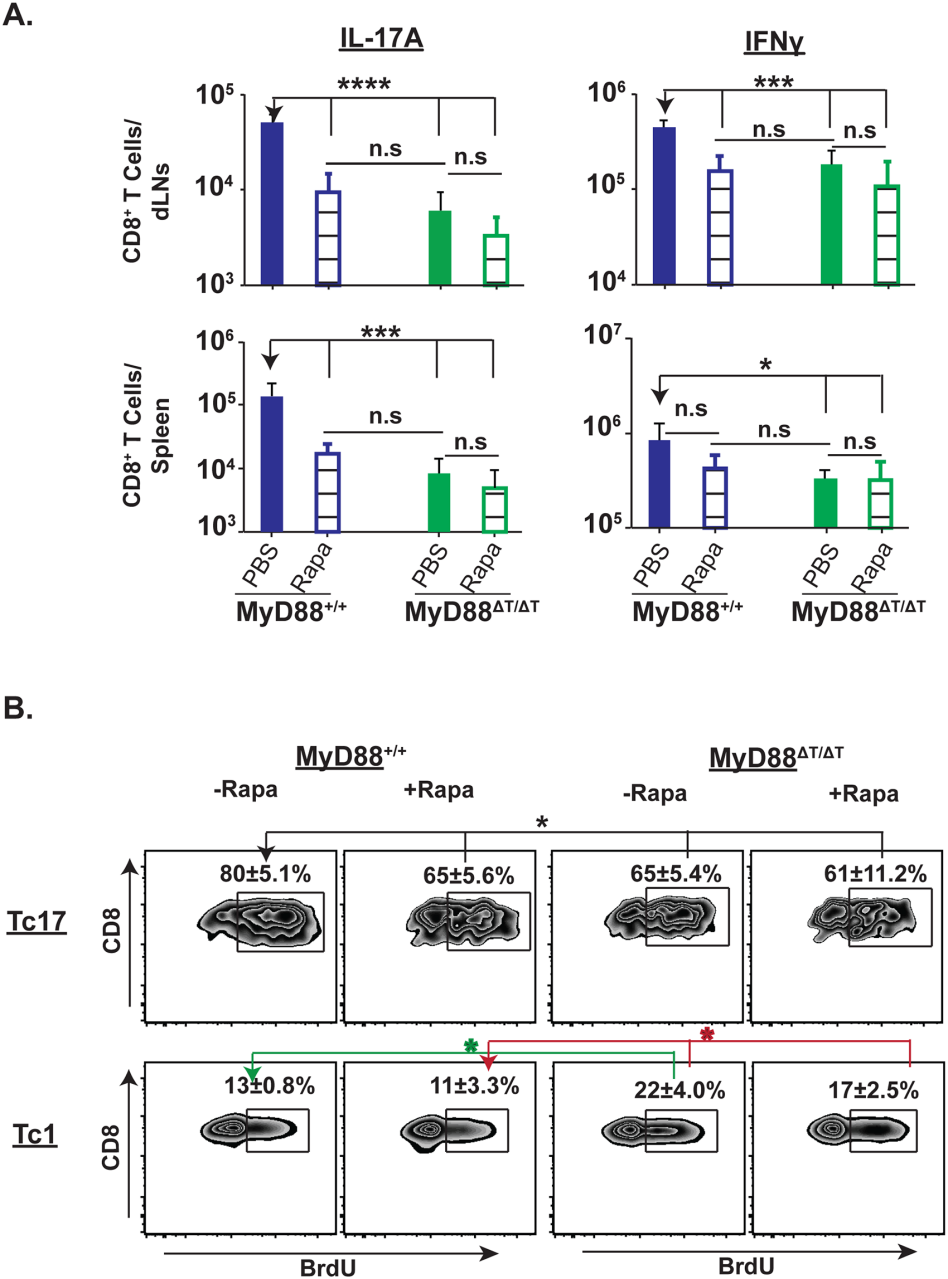
Rapamycin treatment blunts Tc17 cell responses and proliferation. Naïve WT and MyD88ΔT mice were CD4^+^ T cell depleted and vaccinated with strain #55. Rapamycin (~2 μg/mouse) was administered daily from day 4 by the i.p. route. On day 19 post-vaccination, dLNs and spleens were harvested to enumerate percent and total numbers of IL-17 and IFNγ producing CD8^+^ T cells **(A)**. Cohorts of mice were pulsed with BrdU through drinking water from day 7–15. On day 16, dLNs and spleens were harvested to enumerate percent BrdU^+ve^ IL-17A and IFNγ producing CD8^+^ T cells **(B)**. Values are the mean ± SD of 4–7 mice/group. *p≤0.05, ***p≤0.001 and ****p≤0.0001. Data is representative of 2 independent experiments.

mTOR has an important role in the metabolism and functions of both innate and adaptive immune cells [35]. Under Th17 polarization conditions *in vitro*, rapamycin treatment inhibited mTOR, blunting the expression of IL-17a transcript and proliferation of CD4^+^ T cells [36]. We postulated that mTOR mediates the proliferation and/or survival of Tc17 cells in MyD88-sufficient mice, and evaluated the effect of rapamycin treatment on Tc17 and Tc1 responses. Rapamycin treatment significantly blunted the total number and percentage of vaccine-induced Tc17 cells in the dLNs and spleens of wild-type mice, but had no effect in MyD88ΔT mice (Figs 6A and S5). The number of Tc17 cells was similar in rapamycin-treated wild-type mice and MyD88ΔT mice, supporting the requisite role of mTOR for MyD88-dependent Tc17 responses. Rapamycin treatment inconsistently affected Tc1 cell responses in vaccinated wild-type mice, blunting the numbers of Tc1 cells in dLNs, but not in spleen, and leaving the percentage of Tc1 cells unaffected in these organs.

We next asked whether blunted Tc17 responses after rapamycin treatment are due to inhibition of proliferation. Vaccinated mice were pulsed with BrdU and treated with either rapamycin or PBS control. Treatment with rapamycin significantly reduced BrdU^+^ Tc17 cells in wild-type mice, but not MyD88ΔT mice (Fig 6B). In contrast, rapamycin treatment did not significantly affect the proliferation of Tc1 cells in either group of mice (Fig 6B). Thus, MyD88 signaling enhances antifungal Tc17 cell responses by augmenting proliferation of these cells via an mTOR dependent pathway.

### MyD88 signaling activates Akt for Tc17 cell responses and mTOR phosphorylation

**Fig 7:**
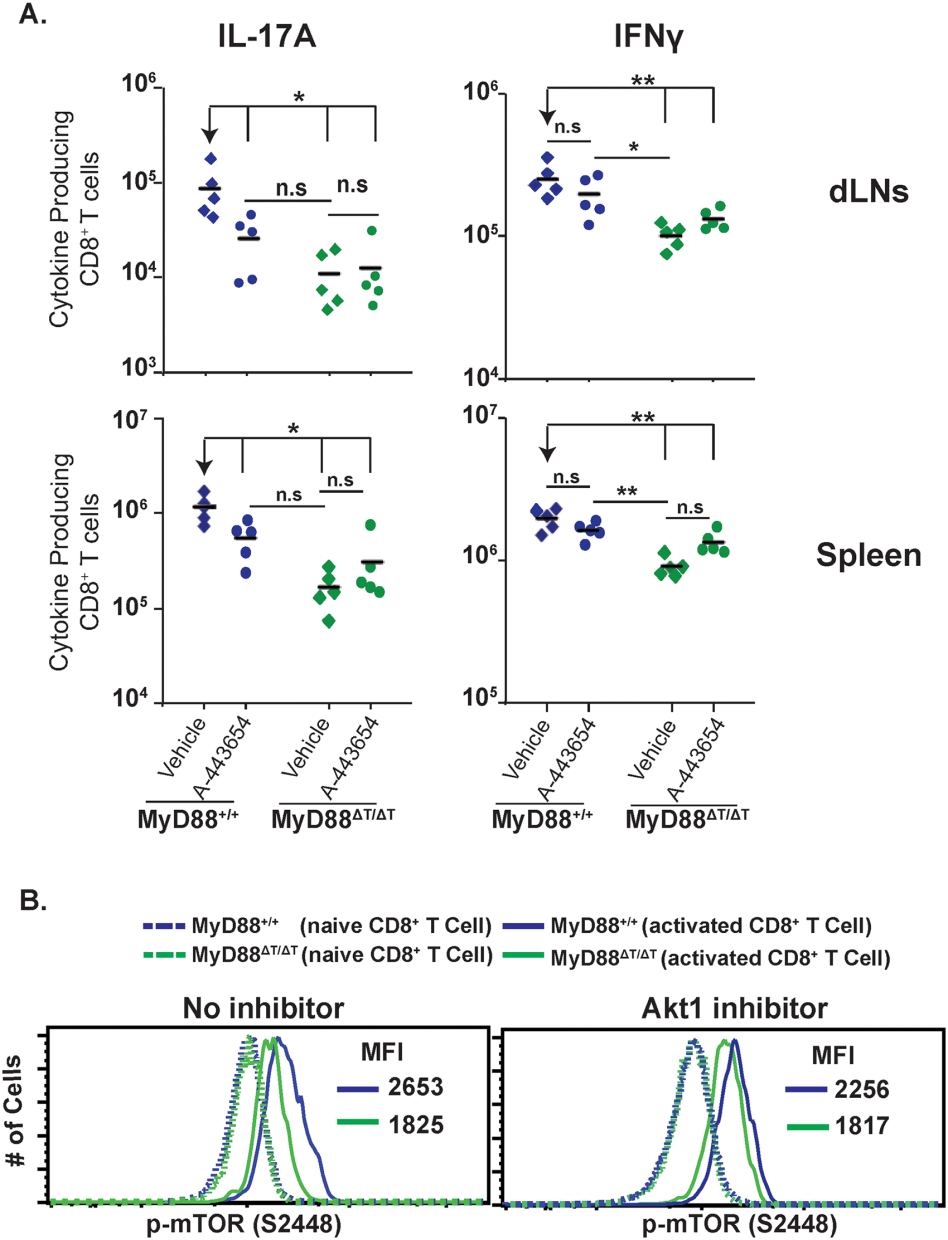
MyD88 requires Akt1 signaling for mTOR activation. **A**. Mice were CD4+ T cell depleted and vaccinated with strain #55. Akt1 inhibitor (A-443654) was administered s.c. from day 4 to 15 post-vaccination. On day 16, dLNs and spleen cells were collected, restimulated and stained for surface markers and intracellular cytokines. Numbers of IL-17A and IFNγ producing CD8^+^ T cells were analyzed by flow cytometry. A diamond represents an individual mouse and the bar is the mean of the group. *p≤0.05 and **p≤0.01. Data is representative of 2 independent experiments. **B**. Purified naive WT and MyD88ΔT OT-I cells were stimulated *in vitro* with anti-CD3 and yeast-stimulated BMDC supernatant. On day 4, supernatant was removed and replaced with culture medium. Cells were rested for 2.5 hours and medium was replaced with yeast stimulated BMDC supernatant either with or without Akt1 inhibitor (1μM) for 1 hour. Cells were surface stained prior to phospho-staining. Values in the histograms represent mean fluorescence intensity (MFI) of p-mTOR. Data in solid lines are from gating on activated cells (CD8^+^CD43^+^CD44^hi^CD62L^lo^), whereas data in dotted lines are from gating on naïve cells (CD8^+^CD43^neg^CD44^lo^CD62L^hi^).

Many kinases can phosphorylate and activate mTOR, including Akt [37]. TLR2 ligation enhanced T-bet expression in CD8^+^ T cells and increased their cytotoxic functions, which were dependent on Akt and mTOR activation [38]. As shown above, mTOR activity is likely modulated by MyD88 signaling. Here, we asked if Akt signaling is required for MyD88-mediated Tc17 responses and mTOR phosphorylation. We first assessed phosphorylation of Akt in CD8^+^ T cells stimulated by yeast *in vitro*. We observed a pronounced increase in phosphorylation of Akt at T308 and S473 sites in the presence of DC supernatant from yeast-stimulated cultures (S6A Fig). We next assessed whether Akt1 is phosphorylated in a MyD88-dependent manner in CD8^+^ T cells activated *in vitro*. We saw higher phosphorylation of Akt1 in wild-type CD8^+^ T cells compared to MyD88ΔT cells consistently at 20, 40 and 60 minutes after activation (S6B Fig). To test the functional role of Akt signaling *in vivo* during vaccination, we inhibited Akt with compound A-443654 [39]. Akt inhibition blunted Tc17 responses in the dLNs and spleen of wild-type mice, but not MyD88ΔT mice (Fig 7A). Conversely, Akt inhibition did not affect Tc1 cell responses in wild-type mice. Instead, Akt inhibition actually increased the numbers of IFN-γ producing cells in MyD88ΔT mice.

The effects of Akt inhibition resembled those of rapamycin treatment, suggesting a possible link between MyD88 activation of mTOR and Akt1 function in regulating downstream Tc17 responses. To test a direct link between them, we analyzed the influence of Akt1 inhibitor on mTOR (S2448) phosphorylation in CD8^+^ T cells. Akt1 inhibited mTOR phosphorylation only in the presence of MyD88; that is, in wild-type CD8^+^ T cells, but in not MyD88ΔTcells, which confirmed that MyD88-dependent activation of mTOR occurred through Akt1 signaling (Fig 7B). Collectively, these results suggest that Akt1 signaling is required for MyD88 dependent Tc17 responses that are mediated through mTOR upon fungal antigen engagement and that these signals are promoted via IL-1 (see section below).

### IL-1R and TLR2, but not IL-18R, signals are required for anti-fungal Tc17 cell responses

**Fig 8:**
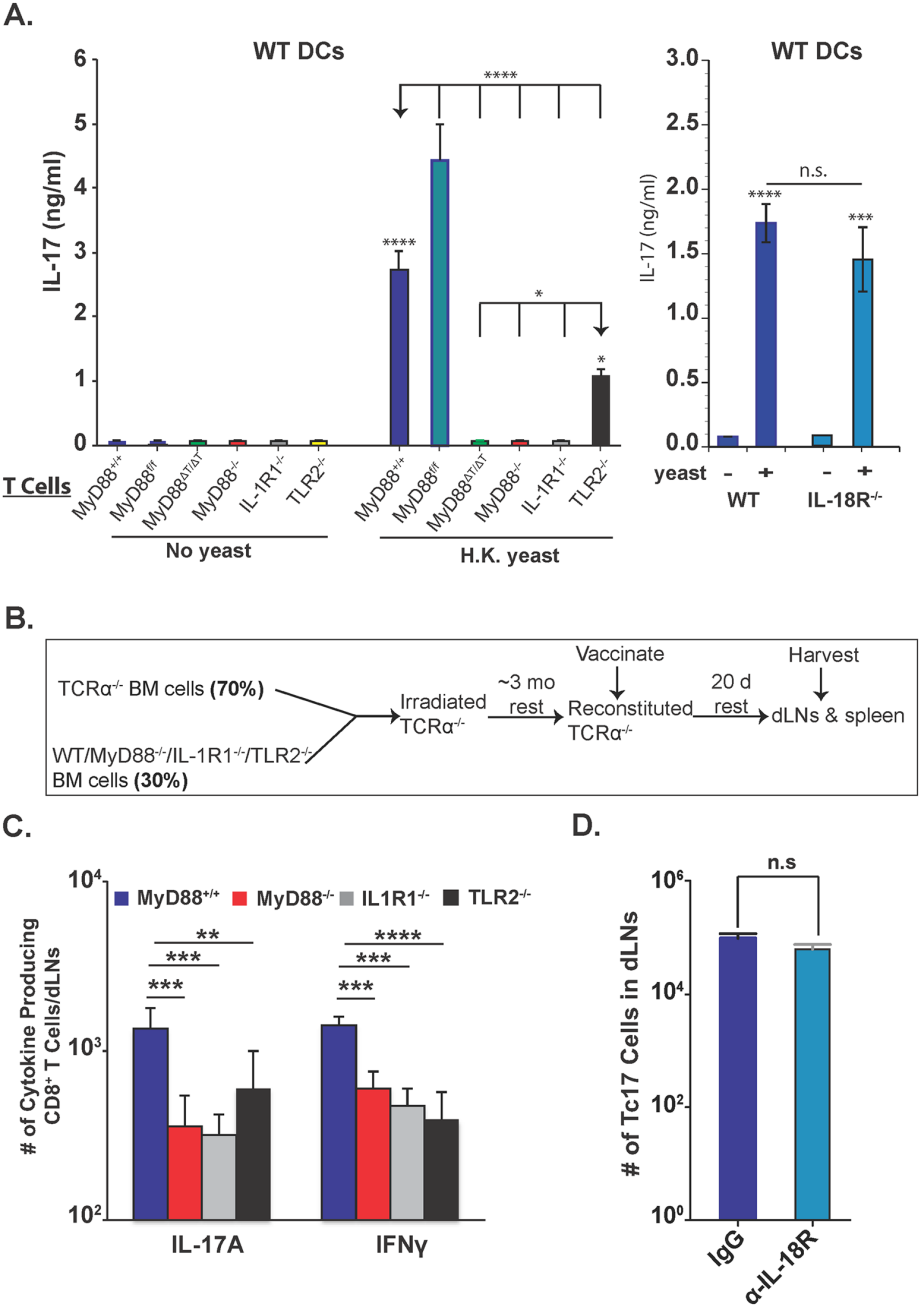
Role of IL-1R, IL-18R and TLR2 on Tc17 cell responses. **A**. Purified naïve CD8^+^ T cells were incubated with WT DCs either with or without heat-killed yeasts for 5 days. Culture supernatants were harvested to quantify IL-17A production by ELISA. Error bars are mean ± SD of triplicate wells. Data are representative of two independent experiments. **B**. Overview of the experimental approach for generation of bone-marrow chimera mice and harvesting of organs from vaccinated mice. **C**. Number of cytokine producing cells in dLNs 20 days post-vaccination. **D**. Number of cytokine-producing cells in the dLNs in either control IgG or anti-IL-18R blocking antibody-treated, vaccinated-mice groups on day 14. CD4^+^ T cells were depleted throughout the experiment. Error bars are mean ± SD of N = 4–7 mice/group. *p≤0.05, **p≤0.01, ***p≤0.001 and ****p≤0.0001.

We previously reported that Tc17 cells are reduced in IL-1R^-/-^ mice [2]. Others have documented TLR2 and IL-18R signaling in CD8^+^ T cells [38,40], although the intrinsic role of IL-1R, TLR2 and IL-18R for Tc17 responses has not been described. We assessed the function of these receptors *in vitro* and *in vivo*. For *in vitro* studies, we purified CD8^+^ T cells and incubated them with wild-type or MyD88^-/-^ BMDCs loaded with yeasts. IL-17A levels were significantly lower in the supernatants from IL-1R1^-/-^, MyD88^-/-^, TLR2^-/-^ and MyD88ΔT vs. wild-type CD8^+^ T cells, but were unaffected for IL-18R^-/-^ CD8^+^ T cells (Fig 8A). The deficit in IL-1R^-/-^ CD8^+^ T cells was similar to the MyD88-deficient groups, and more pronounced than for TLR2 deficient CD8^+^ T cells. Phosphorylation of Akt at T308 and p-mTOR levels in CD8^+^ T cells were enhanced by the addition of either IL-1α or IL-1β or both, but only in the presence of MyD88 (S6C Fig). CD8^+^ T cells incubated with MyD88^-/-^ BMDCs also produced significantly less IL-17A (S7 Fig), which is consistent with an independent, extrinsic contribution.

For *in vivo* studies, we created mixed bone-marrow chimeras using irradiated TCRα^-/-^ mice as a recipient for different donors (Fig 8B), or administered blocking antibody against IL-18R throughout the study. Tc17 and Tc1 cells were both reduced in the dLNs in the absence of T-cell specific MyD88, IL-1R and TLR2 (Fig 8C), but Tc17 cells were unaffected by blockade of IL-18R (Fig 8D). The spleens of chimeric mice revealed more pronounced impairments in Tc17 vs. Tc1 cells (S7 Fig), however TLR2^-/-^ Tc17 cell numbers were not significantly different from wild-type. Thus, IL-1R and TLR2 exert hierarchical contributions to intrinsic MyD88 signaling in Tc17 cells, with the former being most important, and IL-18R signals appears to be dispensable in this model of vaccine-induced anti-fungal Tc17 cells.

## Discussion

Th17 cell responses are essential for immunity against infections including those caused by fungi [9]. AIDS and other immune compromising disorders are associated with increased rates of opportunistic fungal infections due to CD4^+^ T cell lymphopenia [41]. Hence, uncovering residual protective immune cells against fungi is essential for vaccination of *at risk* individuals. We previously showed that in the absence of CD4^+^ T-cell help protective anti-fungal CD8^+^ T cell responses are elicited and maintained as long-lasting memory cells. Tc17 cells are indispensible for this vaccine-induced fungal immunity [2]. The extrinsic cytokine signals required for differentiation of Tc17 (and Th17) cells have been characterized, and include TGFβ, IL-6, IL-21, and IL-23. However, the role of T cell intrinsic signals including MyD88 and upstream TLRs and IL-1R family members for Tc17 (and Tc1) responses during immunity to infection has been less clear. Here, we document a requisite role for intrinsic MyD88 in Tc17 cell responses and fungal vaccine immunity. We also show that under the same ‘inflammatory milieu’, intrinsic MyD88 signals are indispensable for Tc17 cell responses, whereas Tc1 cells are less affected in the absence of these signals.

MyD88 deficiency enhances susceptibility to infections caused by viruses, bacteria, parasites and fungi, but its contribution to resistance varies depending upon the pathogen. MyD88 is essential for innate immunity and resistance without affecting CD8^+^ T cell responses during *Trypanosoma* infection [42]. In contrast, MyD88 has a cell-intrinsic role for CD8^+^ T cell responses during lymphocytic choriomeningitis and vaccinia infections, where Tc1 responses are compromised in its absence [31,43,44]. During experimental toxoplasmosis, T-cell-intrinsic MyD88 deficiency severely affects Th1 responses and impairs resistance [28]. Our study unveils a critical role for intrinsic MyD88 function in CD8^+^ T cells during vaccine immunity against fungal pneumonia; its absence leads to a profound deficit of Tc17 responses in the lung.

We explored mechanisms underpinning MyD88 dependent, intrinsic control of Tc17 responses. After antigen engagement, T cell expansion is the net result of effector T cell proliferation and death. Several modes of cell intrinsic MyD88 action are possible. Lack of intrinsic MyD88 signaling during viral infection enhanced apoptosis of effector CD8^+^ T cells (despite normal Bcl-xL expression) without affecting proliferation [31]. In contrast, intrinsic MyD88 was required to sustain proliferation of effector Tc1 cells in a model of protracted viral infection [30]. Our findings suggest that intrinsic MyD88 is required for sustained proliferation of Tc17 cells, but not Tc1 cells. Our studies also show that MyD88^-/-^ CD8^+^ T cells were not prone to apoptosis and that both Tc17 and Tc1 cells displayed similar levels of active-caspase3, Annexin V and Bcl-xL expression. Bcl-2 levels were also not influenced by MyD88 expression in Tc17 cells, however Bcl-2 levels were lower in Tc17 cells than in Tc1 cells. The relevance of this finding is unclear, but our prior work showed that Tc17 cells portend long-term memory and display stem-cell like features [2].

Akt signaling is integral for T cell activation and expansion [45]. T cell differentiation may involve combinatorial signals that naïve T cells receive under a ‘micro-inflammatory milieu’, but the role of TCR signaling in regulating T cell responses via MyD88-Akt for Tc17 cell responses is poorly understood. Intrinsic MyD88 signals are known to boost functional avidity of IFNγ^+^ CD8^+^ T cell responses during vaccinia vaccination by reducing the activation threshold [46], whereas our data show that these signals augment Tc17 responses more than Tc1 responses. We also found that Akt1 signals were critical for boosting Tc17, but not Tc1 responses, suggesting the hypothesis that low avidity CD8^+^ T cells require MyD88 signals to augment Tc17 responses whereas high avidity Tc1 cells may not require augmented Akt signaling. This idea is in line with data from Th17 cells where low-strength T cell activation promotes their phenotype [47]. Our *in vivo* results support this premise since Tc1 cells were unimpaired or even augmented in the presence of Akt1 inhibitor.

mTOR, a key metabolic sensor, is chiefly activated by PI3K-Akt pathway in T cells [35]. To our knowledge the role of MyD88 in Akt-mTOR regulation of Tc17 cell responses has not been defined, although ligation of TLR2 has been shown to enhance T-bet and Tc1 cell responses in a manner dependent on Akt and mTOR [38]. mTOR activity has been linked to Th17 cell responses by enhancing HIF-1α expression, Stat3 phosphorylation, RORγt translocation, and cell proliferation [48]. We show here that pharmacological inhibition of mTORC1 reduced Tc17 cell, but not Tc1 cell proliferation in a MyD88-dependent manner. We also found that MyD88 deficiency did not affect RORγt levels, similar to a report on Th17 cell polarization [36]. That study, which involved *in vitro* Th17 polarization, showed that IL-1 signaling was required for the expression of IL-23R and together they enhanced mTOR activity to promote Th17 cell responses. Other cytokines including IL-6, IL-21 and Il-23 also can augment the expression of IL-23R [49]. Our studies suggest that MyD88 signals influence mTOR through Akt to enhance Tc17 cell responses, and that MyD88 signaling may function independent of IL-23 signaling through Akt. Nevertheless, IL-23 signaling in Th17/Tc17 cells may enhance Akt-mTOR signaling via Jak2 [50]. Further studies are needed to address whether IL-23 integrates MyD88 signaling downstream of mTOR. One possible mechanism is that MyD88-Akt-mTOR may bolster stat3 function [48,51] that is activated by IL-6, IL-21 and IL-23. Rapamycin treatment can also affect innate immunity by mechanisms that may, in turn, affect Tc17 cell responses [52]. Our *in vivo* work suggested that Rapamycin treatment of vaccinated mice chiefly and selectively affected intrinsic MyD88 signaling for Tc17 cell responses, in view of the insignificant effect on Tc17 responses in MyD88ΔT mice.

Accumulating evidence suggests that IL-1 is required for both systemic and mucosal Th17 responses [53]. IL-1 has pleotropic effects on both innate and adaptive immunity and the lack of IL-1 enhances the susceptibility to bacterial, viral and fungal infections [54]. IL-1R^-/-^ mice are vulnerable to coccidioidomycosis and blastomycosis, and IL-1 administration was shown to enhance fungal vaccine immunity in a manner that required IL-17R signaling [51,55]. Administration of IL-1 enhanced the expansion and function of CD8^+^ T cells [55] and IL-1R^-/-^ mice had reduced CD8^+^ T cell responses, IFN-γ production and viral clearance [56], although the cell-intrinsic role of IL-1R signaling for CD8^+^ T cell responses was not explored. Our study here shows that intrinsic IL-1 signaling sharply affects Tc17 cell responses. This differing impact of IL-1 on Tc17 vs. Tc1 responses in the two studies may be due to the model system where differentiation towards Tc1 cell responses is favored and exogenous IL-1 just augmented the responses by increasing T-bet and activating mTOR [38].

Ligands for TLR2 influence the polarization of Th17 cells *in vitro* and development of EAE in mice [29]. Among the many ligands known for TLR signaling, fungi display zymosan, phospholipomannan, O-linked mannans, and DNA, which are recognized by TLR2, TLR4 and TLR9, respectively. While impaired MyD88 signaling reliably enhances susceptibility to numerous fungal infections, the absence of individual TLRs shows varying results, perhaps due to impaired IL-1R family signaling in MyD88^-/-^ mice or due to compensation by other TLRs [9]. While a T-cell intrinsic role was not explored, IL-1R but not TLR2 signaling was essential for Th17 responses during *Coccidioides* infection [51]. In an *in vitro* model, a TLR2 agonist was shown to enhance T-bet expression in CD8^+^ T cells by activating Akt and mTOR [38]. Here, we observed that intrinsic TLR2 signaling was essential for Tc17 as well as Tc1 responses. Differences among these studies may be due to the model, fungal strain, T cell type or compensation by other receptors. Our data also suggested that TLR2 was only essential for initial priming in the skin dLNs, but not in the spleen, where exuberant circulating IL-1 may compensate the defect for Tc17 responses.

Collectively, we show that intrinsic MyD88 signals are required for anti-fungal vaccine immune responses in vulnerable CD4+ T cell deficient hosts through sustained proliferation and preferential expansion of Tc17 cells, which is dependent on Akt and mTOR. Our study therefore identifies unappreciated targets for augmenting adaptive immunity against pathogenic fungi. Our findings are important for designing vaccines against fungal infections in *at risk* individuals with CD4^+^ T cell defects and for immunotherapeutic intervention during infection and possibly autoimmune disorders.

## Methods

### Ethics statement

Animal procedures were done in accordance with the recommendations in the Guide for the Care and Use of Laboratory Animals of the National Institutes of Health. Care was taken to minimize animal suffering. The work was done with the approval of the IACUC of the University of Wisconsin-Madison. The IACUC protocol number for the study is M00969.

### Mice

Seven- to eight-week-old C57BL/6 (WT) were purchased from the National Cancer Institute. Inbred strains of mice on the C57BL/6 background were purchased from Jackson Laboratories and included Thy1.1 allele carrying congenic B6 mice strain B6.PL-Thy1^a^/Cy (Stock 000406), Ly5.1 allele carrying congenic B6 mice strain B6.SJL-*Ptprc* ^*a*^ *Pep3* ^*b*^ */*BoyJ (stock 002014), Il1r1^-/-^ B6.129S7-*Il1r1* ^tm1Imx^/J mice (stock 003245), B6.129-*Tlr2* ^*tm1Kir*^ /J (stock004650), Il18r1^-/-^ B6.129P2-*Il18r1* ^*tm1Aki*^/J, B6-Tg (TcraTcrb)1100Mjb/J (003831), B6.129P2(SJL)-*Myd88* ^*tm1Defr*^ /J (Stock 008888), and B6.129P2(SJL)-*Myd88* ^*tm1*.*1Defr*^ /J (Stock 009088). Thy1.1^+^ OT-I Tg mice were generated by crossing the Thy1.1 allele carrying strain with the OT-I Tg strain. Breeding pairs of T-cell specific MyD88^-/-^ (MyD88ΔT) mice were a kind gift from Laurence Turka. Congenic OT-I Tg-MyD88ΔT (Thy1.1) mice were generated by crossing OT-I Tg (Thy1.1) and MyD88ΔT mice. Mice were housed and cared for according to guidelines of the University of Wisconsin Animal Care Committee, who approved all aspects of this work.

### Fungi

Wild-type virulent *B*. *dermatitidis* strain 26199 was purchased from ATCC. An isogenic attenuated mutant lacking BAD-I (strain #55) and recombinant #55 strain carrying the OT-I epitope SIINFEKL were also used for vaccination (below). Isolates were maintained as yeast on Middlebrook 7H10 agar with oleic acid-albumin complex (Sigma-Aldrich, St. Louis, MO) at 39°C.

### Vaccination, infection and CD4^+^ T cell depletion

Vaccination of mice with specific strains of fungus has been described elsewhere [1]. Briefly, ~10^5^ cfu of attenuated strain #55 was inoculated subcutaneously (s.c.) at each of two sites, dorsally and at the base of tail. For vaccination with recombinant OT-I #55 strain, a total of 10^7^ yeast was used after heat killing at 65°C for 30 min. Pulmonary challenge studies were done with 2x10^3^ yeast of wild-type strain #26199. CD4^+^ T cell depletion was performed by using a weekly dose of 100 μg of GK1.5 mAb (Biovest International Inc. Minneapolis, MN/BioXCell, West Lebanon, NH) given intravenously (i.v.), with an efficiency of >99% depletion [1].

### Adoptive transfers

All adoptive transfers of enriched CD8^+^ T cells were done using BD Biosciences (Palo Alto, CA) or Miltenyi kits (Auburn, CA). Equal numbers of CD8^+^ T cells were used for transfer into cohorts of recipients by the i.v. route.

### IL-18R blocking experiments

For blocking IL-18R, we purchased anti-IL-18R (R&D Systems) and administered 400 μg/mouse on day -1 of vaccination and 300 μg/mouse subsequently every 3 days by the i.v. route.

### Flow cytometry

Organs were harvested and single cell suspension cells prepared using BD biosciences cell strainers and plunger. Cells were subjected to Fc block (BD Biosciences) for 20 min before staining for surface markers with antibodies for 30 min at 4°C. Fluorochrome-labeled anti-mouse antibodies against CD8 (clone 53–6.7), Thy1.1 (clone OX-7), CD45.1 (clone A20), CD27 (clone LG.3A10), IFNγ (clone XMG1.2), TNFα (clone MP6-XT22), IL-17A (clone TC11-18H10), IL-2 (clone JES6-5H4), BrdU Flow kit (Cat 51-2354AK) and Bcl-2 set (Cat 554221) were purchased from BD Biosciences/Pharmingen, whereas, CD44 (clone IM7), GM-CSF (clone MP1-22E9) and ROR gamma (t) (clone AFKJS9) were purchased from eBioscience (San Diego, CA). Rabbit anti-mouse antibodies phospho-Akt (T308) (C31E5E), phospho-mTOR (S2448) (D9C2), phospho-Akt (S473) (D9E) and Bcl-xL (54H6) were obtained from Cell Signaling. Anti-mouse CD43 (clone 1B11) was purchased from Biolegend (San Diego, CA).

### Intracellular cytokine staining

Single cell suspensions were restimulated with anti-CD3 {clone 145.-2C11; 0.1μg/ml) and anti-CD-28 (clone 37.51; 1μg/ml) in the presence of Golgi-stop (BD Biosciences) for 5 hours at 37°C. Cells were first stained for surface markers followed by intracellular cytokines and/or Bcl-2 and Bcl-xL staining using BD Perm/Fix kit. Cells were analyzed by flow cytometry using an LSR II instrument.

### Phospho-protein staining

Cells were surface stained prior to fixing and permeabilizing with Phosflow Lyse/Fix buffer and Phosflow Perm/Wash buffer II (BD Biosciences). Cells were then stained with phospho-specific antibodies (Cell Signaling, Danvers, MA) and analyzed by flow cytometry and mean fluorescence intensity was determined.

### *In vivo* proliferation assay

BrdU (MP Biomedicals, Santa Ana, CA) was fed through drinking water at the concentration of 0.8 mg/ml daily on indicated days. At the end of the experiment, the cells were restimulated, stained for surface markers and intracellular cytokines as above. Cells were washed and subjected to a BrdU staining protocol as per the manufacturer’s protocol (BD Biosciences).

### Inhibitor treatments

Stock solutions of rapamycin were diluted in PBS and mice were given 2 μg daily by the intraperitoneal route (i.p.). Akt1 inhibitor, A-443654, was suspended in 0.2% HPMC solution and mice were given 7.5mg/kg/d divided twice daily by the subcutaneous route. A-443654 and Rapamycin were kind gifts from Dr. M. Suresh, University of Wisconsin-Madison.

### Bone marrow chimera experiments

TCRα^-/-^ recipient mice were lethally irradiated (a total of Gy 1100) and transfused with mixed 3x10^6^ bone marrow cells in the ratio of 70% TCRα^-/-^ and 30% of respective donor cells. After a 3-month rest period, mice were used for the experiment.

### *In vitro* stimulation assays

Bone marrow cells were obtained and differentiated into dendritic cells (BMDCs) in the presence of GM-CSF and IL-4 for 6 days. 10^5^ BMDCs were incubated with heat killed yeast of strain #55 at a 1:1 ratio and 10^6^ enriched CD8^+^ T cells were added to the well and incubated for 5 days. Culture supernatants were harvested to quantify IL-17A levels by ELISA. In some experiments, enriched CD8^+^ T cells were cultured for 4 days in the presence of anti-CD3 antibody and BMDCs supernatant that was collected after culturing BMDCs with heat killed yeast for 48 hrs. In some experiments, we used supernatant of BMDCs stimulated with yeast for 48 hours as a source of cytokines for *in vitro* stimulation of naïve CD8^+^ T cells, along with the addition of anti-CD3 antibody.

### Statistics

Statistical analysis was performed using a two-tailed, unpaired Student t test. For statistical analysis for fungal CFUs, a nonparametric Kruskal-Wallis test with Dunns post-test was used to compare unvaccinated vs vaccinated groups and among vaccinated groups. For comparing more than 2 groups, one-way ANOVA was used with Bonferroni post-test correction. A 2-tailed P value of ≤0.05 was considered statistically significant.

## Supporting Information

S1 Fig Intrinsic role of MyD88 for Tc1 and Tc17 responses. Purified CD8^+^ T cells (~10^7^) from naïve WT and MyD88^-/-^ mice were adoptively transferred into naïve TCRα^-/-^mice. Recipients were vaccinated with heat-killed #55 yeast, rested for 4 weeks and challenged with virulent strain #26199. Four days later, lungs were harvested to enumerate cytokine producing CD8^+^ T cells. **A**. Experimental design. Frequency (**B**) and total number (**C**) of cytokine-producing cells. Values are mean ± SD. N = 4–6 mice. *p≤0.05, **p≤0.01, and ***p≤0.001.
(TIFF)

S2 Fig Intrinsic role of MyD88 in antigen-specific anti-fungal Tc17 responses. **(A)**. Naïve CD8^+^ T cells from indicated mouse strains are enriched and cultured with WT BMDCs and stimulated with either no yeast or indicated heat-killed yeast strains #55 or OVA expressing (OT-I) #55 for 3 days. **(B)**. Naïve CD8^+^ T cells were stimulated for 4 days with anti-CD3 antibody plus supernatant collected from BMDCs that had been cultured with killed yeast for 48 hours. IL-17A and IFNγ in culture supernatants was quantified by ELISA. *P≤0.05 and **p≤0.01.
(TIFF)

S3 Fig Kinetics of CD8^+^ T cell responses in WT and MyD88ΔT mice. Naïve mice were vaccinated and tissues were harvested on indicated days. Cells were restimulated and stained with antibodies for surface markers and intracellular cytokines, and analyzed by flow cytometry. **A**. Kinetics of activated CD8^+^ (CD44^hi^) T cell response. The top and bottom panels respectively show the frequency and total numbers of activated CD8^+^ T cells in dLNs and spleen. **B**. Mean fluorescence intensity of IL-17A in IL-17A^+^ CD8 T cells of WT and MyD88ΔT mice on day 15 post-vaccination. Values are mean ± SD of 4–5 mice/group. *P≤0.05.
(TIF)

S4 Fig MyD88 signaling sustains proliferation of Tc17 cells. Mice were vaccinated and treated as described in main Fig 5. Spleens were harvested to analyze for BrdU+ cytokine producing effector CD8^+^ T cells. *P≤0.05. Values are mean ± SD of 4–5 mice/group.
(TIF)

S5 Fig MyD88 signaling is required to augment mTOR mediated Tc17 cell response. Experimental procedure is as described in main Fig 6. Percent cytokine producing cells in dLNs and spleens are analyzed by flow cytometry. *P≤0.05. Values are mean ± SD of 4–7 mice/group. Data is representative of two independent experiments.
(TIF)

S6 Fig MyD88-dependent phosphorylation of Akt and mTOR. Enriched wild-type (WT) and MyD88ΔT CD8^+^ T cells were incubated with either unstimulated control BMDC supernatant or yeast-stimulated BMDC supernatant for 3 days. Cells were washed and replated with complete medium for 3 hours before the addition of fresh stimuli (**A & B**) or of medium with IL-1α, IL-1β or both (100ng/ml) (**C**). After indicated time points, cells were washed and stained for pAkt and mTOR. **A**. pAkt levels in WT CD8^+^ T cells after 60 minutes of incubation with yeast-stimulated BMDC supernatant vs. unstimulated control supernatant. Grey line represents isotype Ab control staining. **B**. pAkt levels in WT vs. MyD88ΔT CD8^+^ T cells cultured with yeast-stimulated BMDC supernatant. **C**. The influence of IL-1 α, IL-1β or both on pAKT levels and on p-mTOR levels in WT vs. MyD88ΔT CD8^+^ T cells. Values indicate the mean fluorescence intensity (MFI). Data in the panels is representative of 2 independent experiments.
(TIF)

S7 Fig Role of IL-1R1, TLR2 and IL-18R signaling for Tc17 responses. **A**. Extrinsic role: enriched naïve WT CD8^+^ T cells were stimulated *in vitro* with either no yeast or yeast along with BMDCs from indicated mouse strains. **B & C**. Numbers of IL-17A producing CD8^+^ T cells in the spleens. *p≤0.05, **p≤0.01, ***p≤0.001 and ****p≤0.0001. Values are mean ± SD of 4–7 mice/group. Data is representative to two independent experiments.
(TIFF)
