# Supplementary figures and images for: Intrinsic MyD88-Akt1-mTOR Signaling Coordinates Disparate Tc17 and Tc1 Responses during Vaccine Immunity against Fungal Pneumonia
Source: PLoS Pathog. 2015 Sep 14;11(9):e1005161. doi: 10.1371/journal.ppat.1005161 (PMC4569330; doi:10.1371/journal.ppat.1005161)

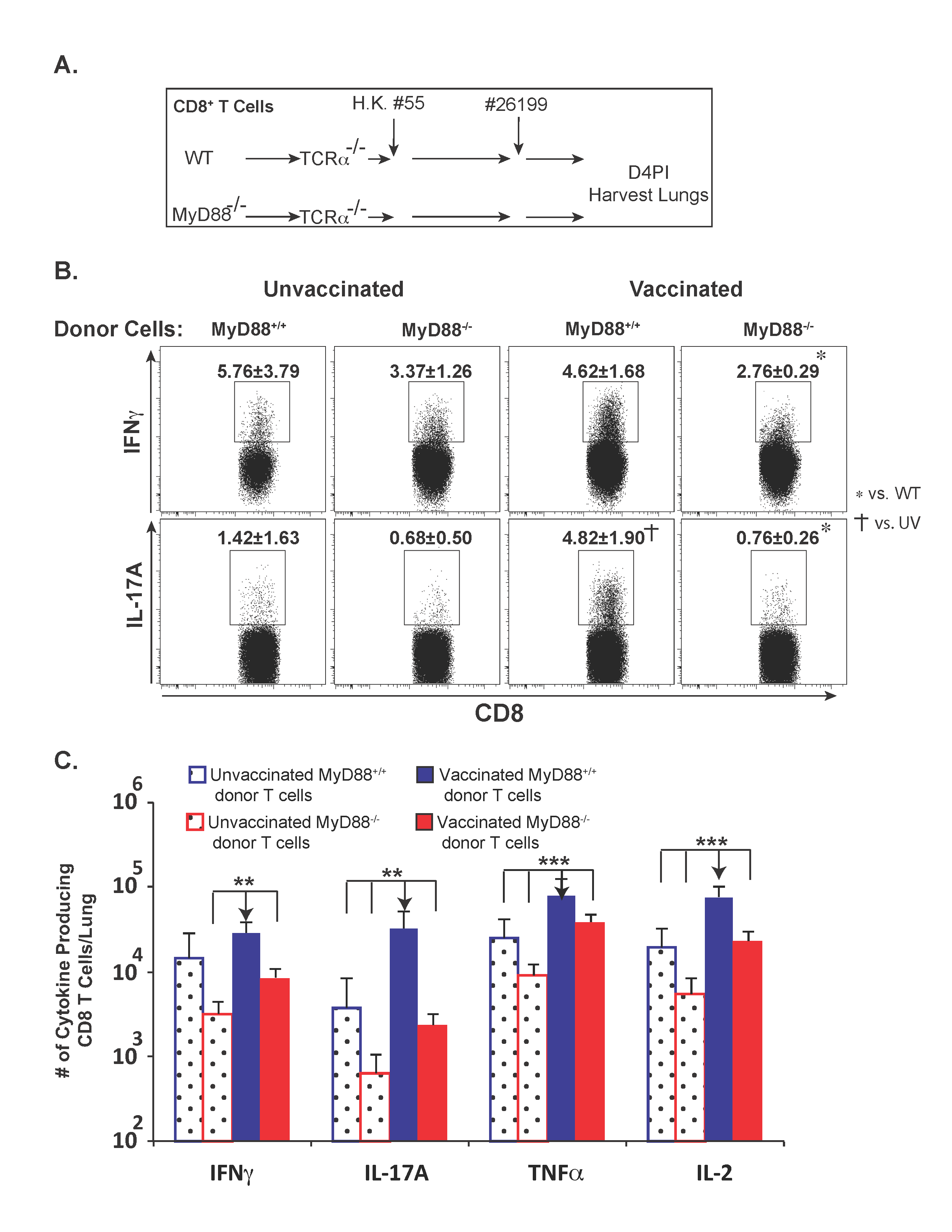

Supplement: S1 Fig — Purified CD8+ T cells (~107) from naïve WT and MyD88-/- mice were adoptively transferred into naïve TCRα-/-mice. Recipients were vaccinated with heat-killed #55 yeast, rested for 4 weeks and challenged with virulent strain #26199. Four days later, lungs were harvested to enumerate cytokine producing CD8+ T cells. A. Experimental design. Frequency (B) and total number (C) of cytokine-producing cells. Values are mean ± SD. N = 4–6 mice. *p≤0.05, **p≤0.01, and ***p≤0.001. (TIFF) [file ppat.1005161.s001.tiff]

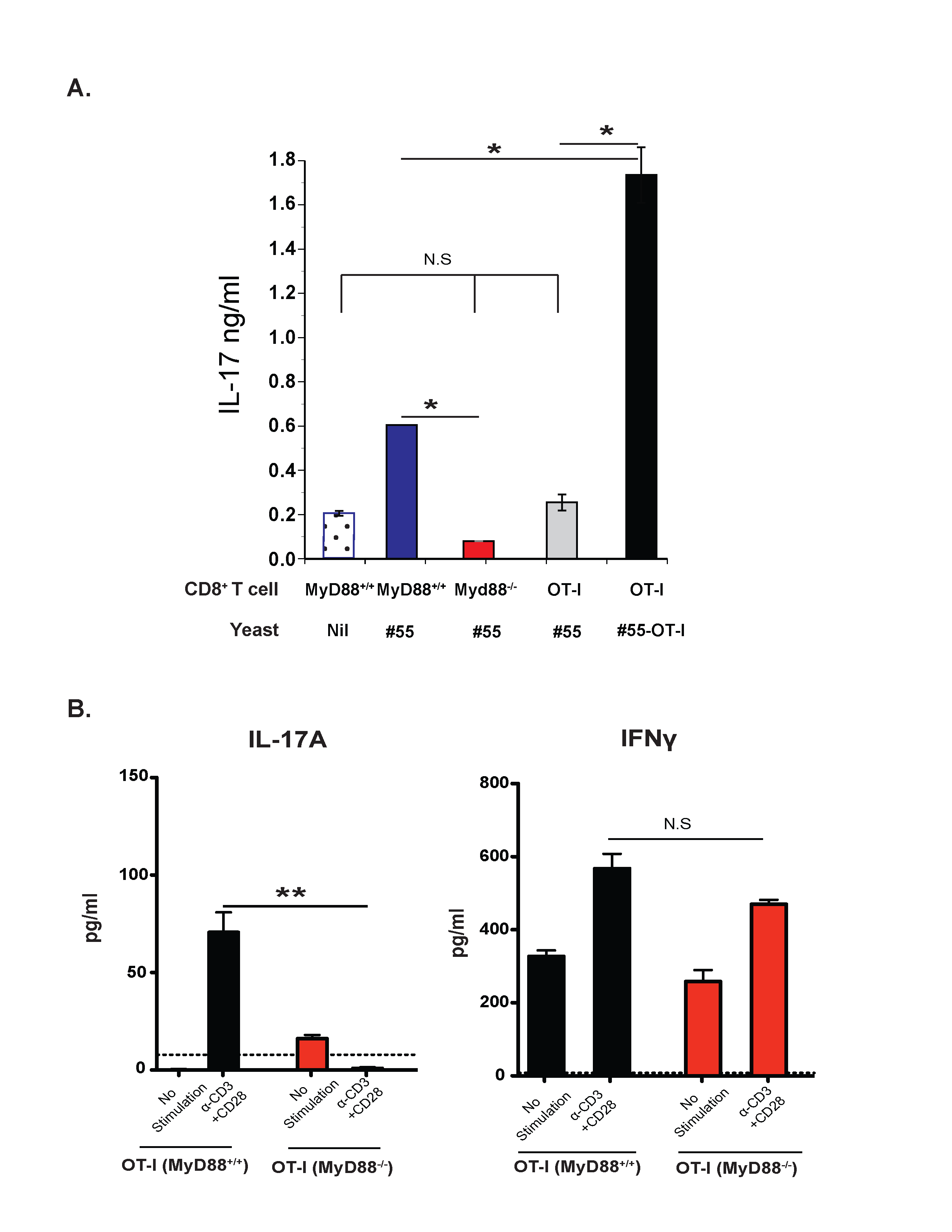

Supplement: S2 Fig — (A). Naïve CD8+ T cells from indicated mouse strains are enriched and cultured with WT BMDCs and stimulated with either no yeast or indicated heat-killed yeast strains #55 or OVA expressing (OT-I) #55 for 3 days. (B). Naïve CD8+ T cells were stimulated for 4 days with anti-CD3 antibody plus supernatant collected from BMDCs that had been cultured with killed yeast for 48 hours. IL-17A and IFNγ in culture supernatants was quantified by ELISA. *P≤0.05 and **p≤0.01. (TIFF) [file ppat.1005161.s002.tiff]

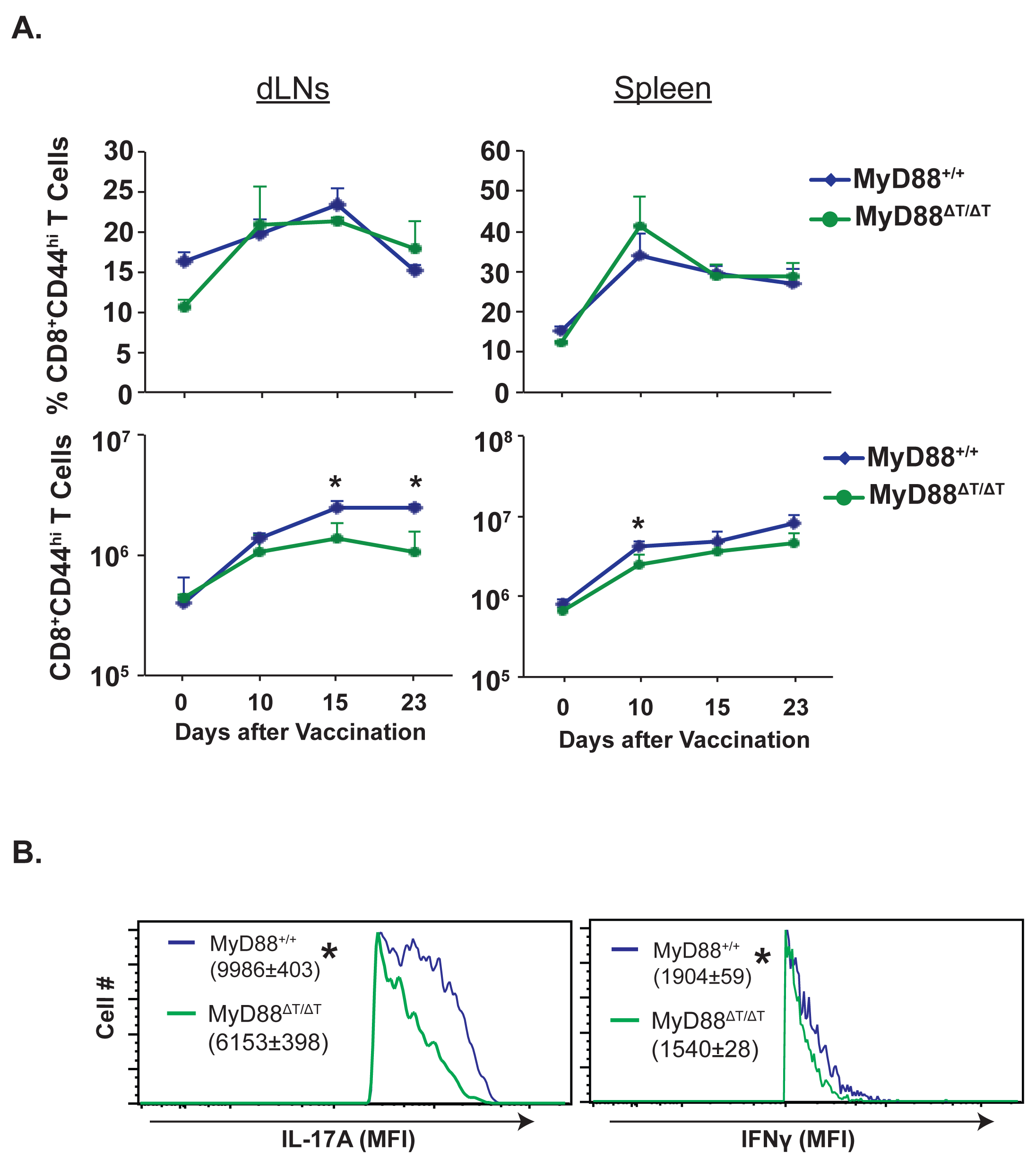

Supplement: S3 Fig — Naïve mice were vaccinated and tissues were harvested on indicated days. Cells were restimulated and stained with antibodies for surface markers and intracellular cytokines, and analyzed by flow cytometry. A. Kinetics of activated CD8+ (CD44hi) T cell response. The top and bottom panels respectively show the frequency and total numbers of activated CD8+ T cells in dLNs and spleen. B. Mean fluorescence intensity of IL-17A in IL-17A+ CD8 T cells of WT and MyD88ΔT mice on day 15 post-vaccination. Values are mean ± SD of 4–5 mice/group. *P≤0.05. (TIF) [file ppat.1005161.s003.tif]

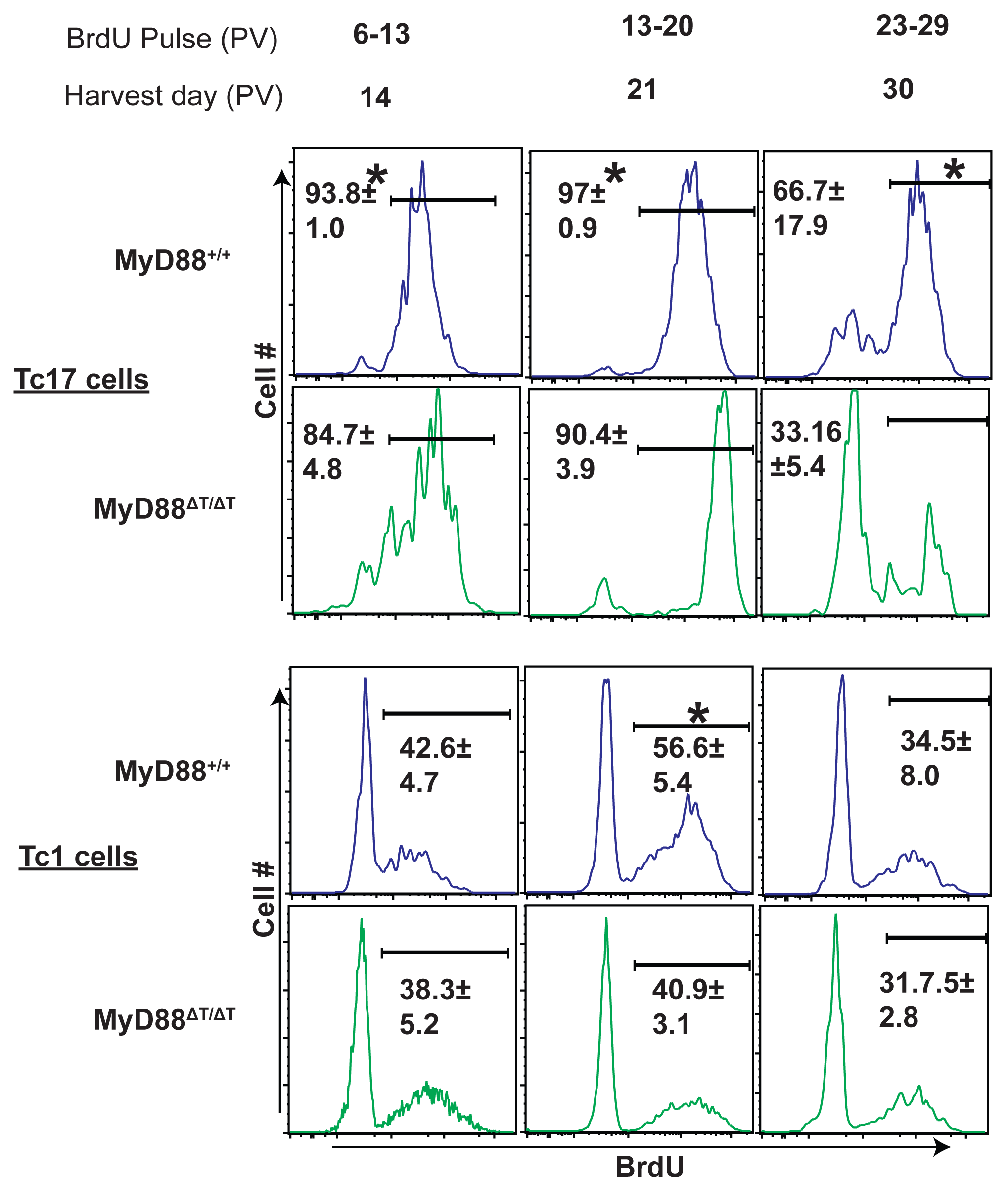

Supplement: S4 Fig — Mice were vaccinated and treated as described in main Fig 5. Spleens were harvested to analyze for BrdU+ cytokine producing effector CD8+ T cells. *P≤0.05. Values are mean ± SD of 4–5 mice/group. (TIF) [file ppat.1005161.s004.tif]

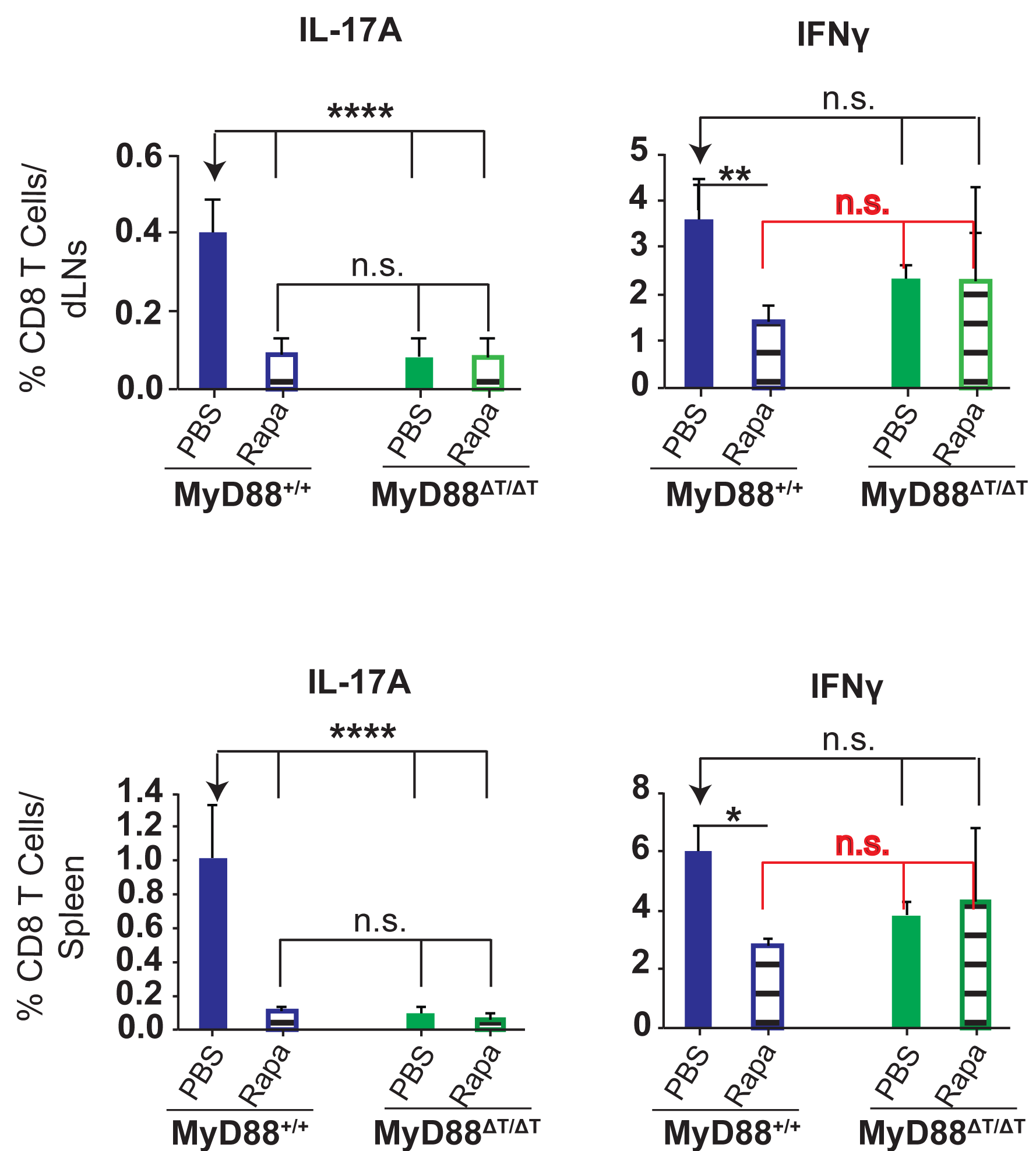

Supplement: S5 Fig — Experimental procedure is as described in main Fig 6. Percent cytokine producing cells in dLNs and spleens are analyzed by flow cytometry. *P≤0.05. Values are mean ± SD of 4–7 mice/group. Data is representative of two independent experiments. (TIF) [file ppat.1005161.s005.tif]

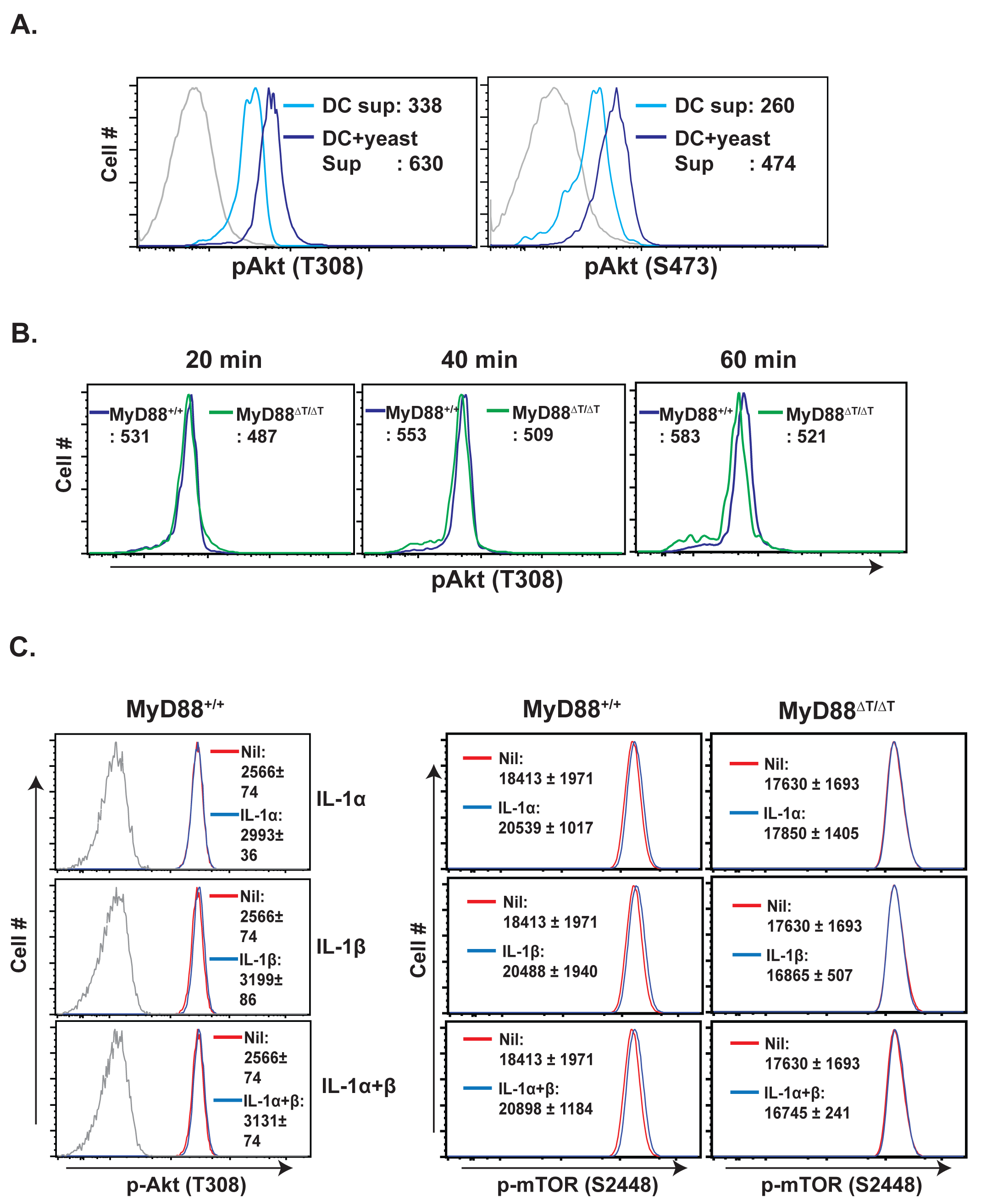

Supplement: S6 Fig — Enriched wild-type (WT) and MyD88ΔT CD8+ T cells were incubated with either unstimulated control BMDC supernatant or yeast-stimulated BMDC supernatant for 3 days. Cells were washed and replated with complete medium for 3 hours before the addition of fresh stimuli (A & B) or of medium with IL-1α, IL-1β or both (100ng/ml) (C). After indicated time points, cells were washed and stained for pAkt and mTOR. A. pAkt levels in WT CD8+ T cells after 60 minutes of incubation with yeast-stimulated BMDC supernatant vs. unstimulated control supernatant. Grey line represents isotype Ab control staining. B. pAkt levels in WT vs. MyD88ΔT CD8+ T cells cultured with yeast-stimulated BMDC supernatant. C. The influence of IL-1 α, IL-1β or both on pAKT levels and on p-mTOR levels in WT vs. MyD88ΔT CD8+ T cells. Values indicate the mean fluorescence intensity (MFI). Data in the panels is representative of 2 independent experiments. (TIF) [file ppat.1005161.s006.tif]

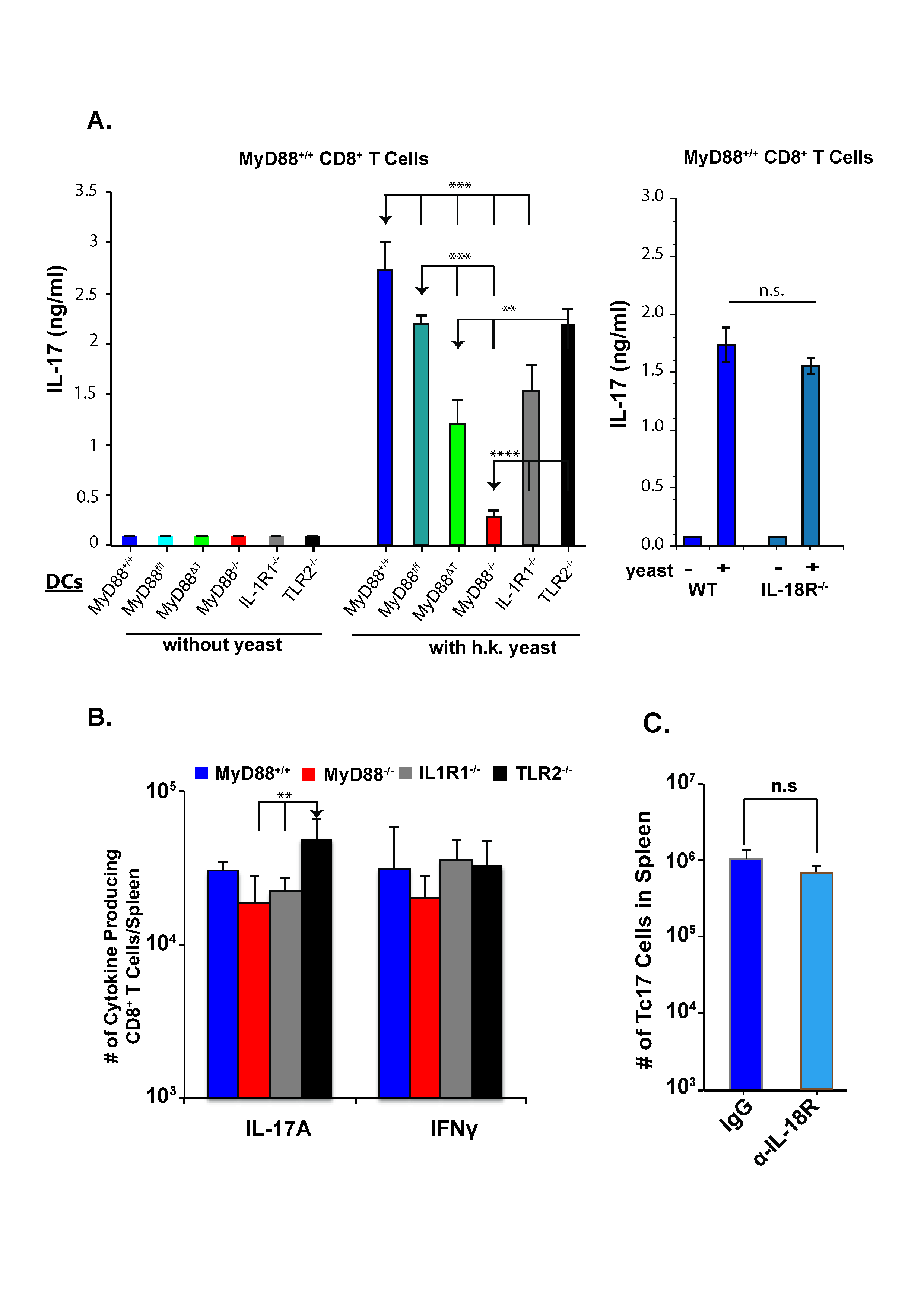

Supplement: S7 Fig — A. Extrinsic role: enriched naïve WT CD8+ T cells were stimulated in vitro with either no yeast or yeast along with BMDCs from indicated mouse strains. B & C. Numbers of IL-17A producing CD8+ T cells in the spleens. *p≤0.05, **p≤0.01, ***p≤0.001 and ****p≤0.0001. Values are mean ± SD of 4–7 mice/group. Data is representative to two independent experiments. (TIFF) [file ppat.1005161.s007.tiff]
